# Cardiovascular, carcinogenic and reproductive effects of nicotine exposure: A narrative review of the scientific literature

**DOI:** 10.12688/f1000research.20062.2

**Published:** 2020-01-09

**Authors:** Leonie R. Price, Javier Martinez

**Affiliations:** 1 Scientific and Regulatory Affairs, Japan Tobacco International, Genève, Genève, 1202, Switzerland

**Keywords:** Nicotine; Electronic Nicotine Delivery Systems, Cardiovascular Diseases, Carcinogenesis, Fetal Development, Fertility, Acute Toxicity, Nicotine Replacement Therapy

## Abstract

The emergence of new tobacco heating products and electronic nicotine delivery systems (ENDS) is changing the way humans are exposed to nicotine. The purpose of this narrative review is to provide a broad overview of published scientific literature with respect to the effects of nicotine on three key health-related areas: 1) cardiovascular risk, 2) carcinogenesis and 3) reproductive outcomes. These areas are known to be particularly vulnerable to the effects of cigarette smoke, and in addition, nicotine has been hypothesized to play a role in disease pathogenesis. Acute toxicity will also be discussed.

The literature to February 2019 suggests that there is no increased cardiovascular risk of nicotine exposure in consumers who have no underlying cardiovascular pathology. There is scientific consensus that nicotine is not a direct or complete carcinogen, however, it remains to be established whether it plays some role in human cancer propagation and metastasis. These cancer progression pathways have been proposed in models *in vitro* and in transgenic rodent lines *in vivo* but have not been demonstrated in cases of human cancer.

Further studies are needed to determine whether nicotine is linked to decreased fertility in humans. The results from animal studies indicate that nicotine has the potential to act across many mechanisms during fetal development. More studies are needed to address questions regarding nicotine exposure in humans, and this may lead to additional guidance concerning new ENDS entering the market.

## Introduction

Newly developed tobacco and nicotine-containing products such as e-cigarettes are being widely accepted by consumers. In order to analyze any potential pathological roles of nicotine, there is a need to consider it in both isolation and within the new landscape of emerging tobacco heating products and electronic nicotine delivery systems (ENDS). Various reviews of published scientific literature and Public Health statements have concluded that despite its addictive nature, nicotine is not a major contributing factor to diseases associated with tobacco smoking. ( Benowitz *et al*., 2016 ; Gottlieb & Zeller, 2017; Royal College of Physicians, 2016; Surgeon General, 2014). Although much investigation has already been carried out, nicotine research continues to be an active field of study. This review will provide an overview of the research up to February 2019, giving insight into the roles of nicotine in the development of acute toxicity, and discuss whether nicotine may adversely affect health in three specific areas: carcinogenesis, cardiovascular risk and reproduction. These areas are known to be particularly vulnerable to the effects of cigarette smoke, and in addition, nicotine has been hypothesized to play a role in disease pathogenesis.

Extensive analysis into nicotine as a pharmacological molecule has already been carried out ( Benowitz, 1996; Mishra *et al*., 2015; and studies reviewed in, e.g., Baraona *et al*., 2017 and West, 2017). This review will attempt not to duplicate existing publications, and instead will focus on the potential effects of nicotine within the context of emerging products and nicotine replacement therapies.

## Search criteria

PubMed was used to carry out literature searches from 2009 to February 2019 with the following search terms: nicotine carcinogenesis, nicotine replacement therapies cancer, nicotine cardiovascular, nicotine acute toxicity cardiovascular, nicotine acute toxicity, chronic exposure to nicotine, nicotine thrombosis, nicotine pregnancy outcomes, nicotine pregnancy, and nicotine fertility.

Studies citing tobacco smoking nicotine exposure were either excluded or addressed cautiously as appropriate. The most recent studies and those carried out in human participants were prioritized where possible, although references to animal and cell studies were necessary, given some lack of studies in humans. Relevant studies and studies prior to 2009 referenced in the returned papers were included. Additional relevant studies from JTI databases were also added. It should be noted that this study was not designed as a systematic review. For full search results, methodology, exclusion criteria, and flow diagram, please see supplementary methods (Extended data ( Price & Martinez, 2019)).

After carrying out the PubMed search and screening, it became clear that many relevant and important papers had been omitted. For this reason, the PubMed search was regarded as the ‘starting point’ for literature inclusion and did not provide the majority of the literature cited. A total of 33 studies were included from the PubMed search, 31 studies were sourced from the references of these studies and a further 58 studies were included from the JTI databases. We cannot exclude the possibility that further relevant studies may not have been included.

## Acute nicotine toxicity

The symptoms of nicotine poisoning, or toxicity are well known and have been characterized through case studies ( Franke & Thomas, 1936; Paik *et al*., 2018), as well as the study of green tobacco sickness (GTS) ( Gehlbach *et al*., 1974). GTS is the manifestation of acute nicotine toxicity typically identified in tobacco workers who have transdermally absorbed high levels of nicotine from handling tobacco leaves. The symptoms include nausea, vomiting, headache, abdominal cramps, breathing difficulty, abnormal body temperature, pallor, diarrhea, chills, fluctuations in blood pressure and heart rate, sweats and increased salivation. It has been demonstrated that the concentration of salivary cotinine, a primary metabolite of nicotine, correlates with the four main symptoms of GTS (headache, nausea, vomiting and dizziness) ( Saleeon *et al*., 2016). The acute symptoms of GTS pass as nicotine is metabolized, however, the long-term effects of GTS are unknown. Within the tobacco-handling work force, multiple GTS incidences may occur in the same individual, and therefore, further research is essential to understand whether there may be any long-term health impact as a result of acute nicotine toxicity.

Current safety databases state that the lethal dose of ingested nicotine in adults is 60 mg ( Cameron *et al*., 2014), which is equivalent to the amount found in approximately five cigarettes ( Mayer, 2014). Despite the low lethal dose and high availability of nicotine in various forms, there are relatively few cases of fatal overdose reported. Conversely, there are many reports of survival after significantly higher exposures ( Solarino *et al*., 2010). After a careful examination of historical reports by Mayer, it seems that the 60 mg dose was derived from self-experimentation carried out in 1857 by Reil, which does not appear to be highly reliable, but is still highly relied upon ( Mayer, 2014). In the intervening period, the 60 mg threshold has become deeply rooted, and therefore, neither questioned nor updated.

Symptoms of acute toxicity have been demonstrated in studies assessing the effects of nicotine replacement therapies (NRTs). In a study investigating the cardiovascular effects of transdermal nicotine patches (discussed in more detail later), participants were administered nicotine using up to three patches equating to 63 mg nicotine. The authors had to change their study protocol because in the highest dose group, nausea and vomiting were experienced 2–4 hours after patch application, corresponding to peak plasma nicotine concentration. For this reason, patches had to be applied at staggered intervals to avoid the toxic effects ( Zevin *et al*., 1998).

Non-smokers often report mild symptoms of nicotine toxicity after exposure to very low levels (2–5 mg), but resistance to the symptoms develops rapidly on repeat exposure and varies extensively between individuals ( Solarino *et al*., 2010).

Since ENDS were introduced to the market, the number of reported incidences of nicotine poisoning have increased in both Europe ( Vardavas *et al*., 2017) and the USA ( Chatham-Stephens *et al*., 2016; Mowry *et al*., 2015). The route of exposure for the majority of these cases were ingestion, inhalation, ocular or dermal, which occurred predominantly in children under the age of five ( Chatham-Stephens *et al*., 2016). Interestingly, Vardavas *et al.* note that “The number of incidents reported to national poison centers were not proportional to either the country’s population or the prevalence of e-cigarette use”. To add context, the Chatham-Stephens paper reported that calls to USA poison centers for nicotine-related incidents peaked in April 2014, with 401 records. For 2014, USA poison centers received an average of 180,428 calls per month related to human poison exposures ( American Association of Poison Control Centers, 2014). Of these, incidents involving cosmetics and cleaning substances were the most common. It should also be noted that calls to poison centers do not capture all incidents, and additionally, do not necessarily represent the occurrence of real poisoning. These data give an indication only. Overall, the reports indicate that acute toxicity from nicotine is highly unlikely to occur when ENDS are used as intended by adults.

## Cardiovascular effects

**Figure 1.**
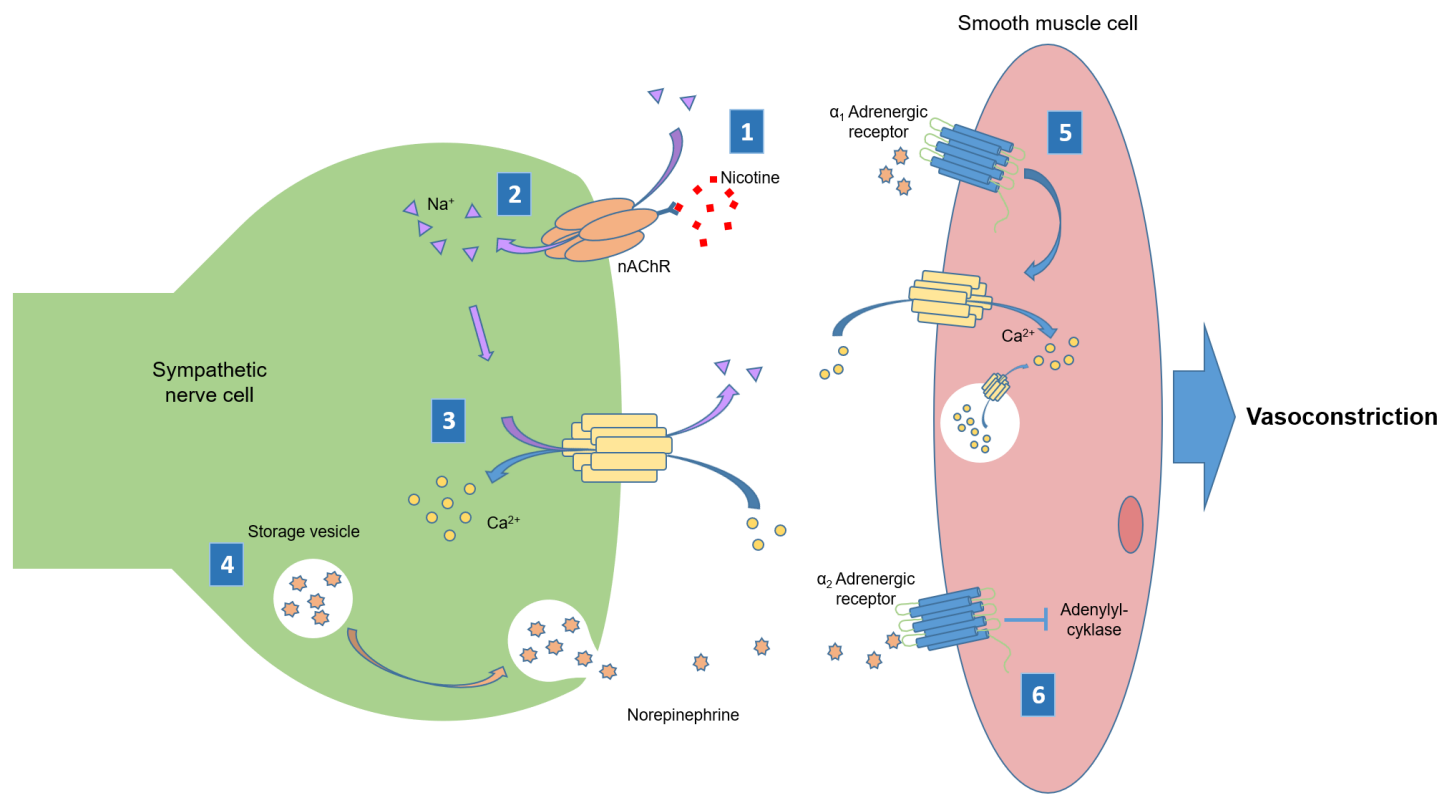
Nicotine stimulation of sympathetic vasoconstriction. 1) Nicotine binds to the nicotinic acetylcholine receptor (nAChR) causing a conformational change. 2) Sodium ions (Na ^+^) enter the cell through the central pore of the nAChR. 3) Ion exchange leads to influx of calcium ions (Ca ^2+^) into the cell causing depolarization. 4) Depolarization stimulates exocytosis of stored norepinephrine. 5) Norepinephrine binds to α _1_ adrenergic receptors on the smooth muscle cell surface, stimulating calcium ion influx and release of stored calcium. 6) Norepinephrine also binds to α2 adrenergic receptors on the smooth muscle cell surface, which causes downstream inhibition of adenylyl cyclase activity. Both these mechanisms cause smooth muscle contraction and, consequently, vasoconstriction.

Nicotine primarily acts on the cardiovascular system through stimulation of the sympathetic nervous system leading to release of norepinephrine and increases in heart rate, blood pressure, myocardial contractility and systemic vasoconstriction ( Figure 1) ( Benowitz & Burbank, 2016; Haass & Kübler, 1997). In addition to this mechanism, stimulation of nAchR expressed in autonomic nervous system ganglia may also influence cardiovascular regulation ( Li *et al.*, 2009). Many studies have investigated the relationship between smoking and increased cardiovascular risk, but few investigate nicotine and cardiovascular risk in humans.

### Human studies

Vansickel *et al.* carried out a study in 20 men and women of various racial backgrounds. All the participants smoked and were healthy. Five minutes after six puffing bouts of an e-cigarette, plasma nicotine increased from a baseline of 2.2 ng/ml to 7.4 ng/ml. Plasma nicotine was only significantly increased after four puffing bouts; heart rate was elevated after the first and second but returned to baseline by the third puffing bout. There was no effect on either systolic or diastolic blood pressure ( Vansickel *et al*., 2012). This study indicated that there is little cardiovascular effect experienced after use of an e-cigarette, although it should be noted that the plasma nicotine levels achieved in this study were lower than those observed in cigarette smokers, which typically peak at 15–25 ng/ml. Nicotine is not the only ingredient in e-liquids. It has been reported that other ingredients, such as propylene glycol, may also have an effect on the cardiovascular system ( Chaumont *et al*., 2018); however, an important recent study by Moheimani *et al.* examined the acute sympathetic roles of the nicotine and non-nicotine based constituents in e-liquids. Despite difficulties inducing an increase in plasma nicotine levels in their participants, the authors observed an increase in sympathetic activity and an increase in heart rate in those who smoked an e-cigarette with nicotine versus without or a sham puff. There was no significant difference in systolic, diastolic or mean arterial blood pressures or measures of oxidative stress and inflammation ( Moheimani *et al*., 2017a). The authors conclude that unlike conventional cigarettes, nicotine is the sole ingredient in e-cigarettes responsible for inducing cardiovascular response. Results published by Franzen *et al.* support this finding. The authors showed that vaping an e-cigarette containing 24 ng/ml nicotine led to significant transient increases in systolic blood pressure, diastolic blood pressure and heart rate. In contrast, vaping a nicotine-free e-cigarette led to no change in systolic blood pressure, and transient small decreases in diastolic blood pressure and heart rate ( Franzen *et al*., 2018). In another study, Moheimani *et al.* also found that long-term e-cigarette users showed decreased heart rate variability and increased oxidative stress compared to non-users, both of which are markers for cardiovascular risk. The authors eliminated the possibility of acute nicotine interference by instructing their participants to abstain from using their e-cigarettes for 12 hours prior to testing. They acknowledged that most of their e-cigarette users were former smokers, and this could have had a chronic effect on heart rate variability, although they state that this was unlikely to be responsible for the observations from the study ( Moheimani *et al*., 2017b).

Farsalinos *et al.* showed that smokers with high blood pressure who reduced their smoking by switching to e-cigarettes showed a significant reduction in systolic blood pressure after 1 year. This suggests that the nicotine found in both cigarettes and e-cigarettes was not responsible (at least entirely) for the chronic increased blood pressure found in the participants. There are too many variables in this study to isolate the effects of nicotine alone, including the fact that very few of the participants stopped smoking conventional cigarettes completely ( Farsalinos *et al*., 2016).

A key study investigated cardiovascular parameters, such as blood pressure, in smokers who switched from conventional cigarettes to e-cigarettes exclusively for five days. The authors estimated the nicotine exposures from the e-cigarette group to be similar to those in the conventional cigarette group, based on usage. Nevertheless, a significant reduction in heart rate, and systolic blood pressure was observed in the e-cigarette group ( D’Ruiz *et al*., 2017). The results indicate that nicotine was not responsible for the cardiovascular profile of the participants, as changes were observed despite nicotine exposure remaining similar across all study groups. The authors concluded that other constituents in conventional cigarettes are more likely to cause the damage to cardiovascular health observed in smokers ( D’Ruiz *et al*., 2017). Conversely, when non-smokers smoked a single cigarette and then an e-cigarette one week later, similar vascular effects were observed in response to both products. There was a significant decrease in flow-mediated dilation (FMD) and an increase in markers of endothelial dysfunction after both the cigarette and e-cigarette, although the changes after e-cigarette use were smaller ( Carnevale *et al*., 2016). The authors suggest that nicotine could be the cause of these changes, although the effects of other e-liquid components (such as flavorings, propylene glycol and glycerin), could not be ruled out. Chaumont *et al.* also raised this point in their paper, where they stated, “The pharmacological actions of nicotine make it impossible to distinguish the respective effects of the carriers themselves (propylene glycol and glycerin) and nicotine on the endothelial dysfunction, oxidative stress and arterial stiffness increase they observed”. To address this, the group carried out a study to assess vascular parameters in response to vaping with nicotine, vaping without nicotine and sham vaping in a small group of 25 non-daily smokers. They observed a significant decrease in acetylcholine-mediated vasodilation, as well as an increase in heart rate, diastolic and systolic blood pressure after nicotine vaping *vs.* baseline, for up to 120 minutes after use. An effect was also seen after use of the non-nicotine-containing e-liquid, but this was only significant for heart rate during vaping. It is not clear whether there was a significant difference between nicotine and non-nicotine-containing e-liquids as this statistical comparison was not carried out; the authors compared only to baseline. The vaping protocol used in this study was intense and used high-powered e-cigarette devices. The authors did not measure plasma nicotine concentrations in their participants, but the consumption of e-liquid far exceeded that of “regular vapers’ habits”. Once again, this study looked only at the acute effects ( Chaumont *et al*., 2018); similar acute responses to nicotine-containing e-cigarettes have also been shown in additional studies ( Franzen *et al*., 2018; Ikonomidis *et al*., 2018; Joesbury *et al*., 1998; Vlachopoulos *et al*., 2016; Yan & D’Ruiz, 2015), as well as with NRT ( Johnston *et al*., 2018; Sabha *et al*., 2000).

An alternative model of nicotine exposure is snus, an oral tobacco product used predominantly in Nordic countries. A study carried out in healthy Norwegian men also found increased markers of endothelial dysfunction in habitual snus users, even after a four-hour period of abstinence. A decrease in FMD was observed across the 238 participants who exclusively used snus, with the largest decreases seen in the most inactive participants. Interestingly, those who achieved recommended daily levels for exercise, or were classified as having high physical fitness did not show any decrease in FMD. This suggests an interaction between several cardiovascular pathways as well as the nicotine response ( Skaug *et al.*, 2016).

In the first study of its kind, Polosa *et al.* followed a small group of e-cigarette users who had never smoked (n = 9; six of whom used nicotine-containing e-liquid), over a period of 3.5 years. At baseline, there were no differences between the vapers and their age/sex-matched controls for all parameters except heart rate (vapers had a slightly lower heart rate compared to the controls). After 3.5 years, there were no changes to any of the cardiovascular parameters measured (heart rate, systolic or diastolic blood pressure) in any of the participants, including those who used the nicotine-containing e-liquid. The participants were all young and healthy (average age 26.6 and 27.8 years for the EC users and controls respectively), and therefore were not at high risk of cardiovascular outcomes. The number of participants was also very small; however, this preliminary study into chronic exposure to nicotine-containing e-liquids suggests that the long-term cardiovascular risks in healthy users are limited ( Polosa *et al*., 2017).

Human studies into the cardiovascular outcomes of nicotine consumed by vaping vary significantly in study design and rarely measure the concentrations of plasma nicotine achieved in the study participants. In addition, e-cigarette devices have evolved rapidly since first becoming commercially available in 2007 and nicotine delivery has improved in line with this evolution. Can we, or should we make a decision as to the cardiovascular effects of nicotine in e-cigarettes based on this inconclusive literature? This sentiment was clearly supported by Skotsimara *et al.* in their systematic review and meta-analysis ( Skotsimara *et al*., 2019). The authors assessed all studies that investigated e-cigarettes in conjunction with cardiovascular measures, including pre-clinical ( *in vitro*) and clinical studies. In total, 26 relevant papers were identified, out of an initial search yielding 7491 papers. When the authors carried out a meta-analysis on studies that specifically addressed the acute effects on heart rate associated with e-cigarettes, only 11 studies were eligible for inclusion, and there was significant heterogeneity between them. As expected, careful analysis showed that there was an acute increase in heart rate associated with e-cigarette use (pooled weighted MD = 2.27, 95% CI: 1.64-2.89, p <0.0001). Seven studies were analyzed for acute effects on systolic and diastolic blood pressure: “Electronic cigarette smoking significantly increased systolic blood pressure acutely (pooled weighted MD = 2.02, 95% CI: 0.07 to 3.97, p = 0.042) and diastolic blood pressure (pooled weighted MD = 2.01, 95% CI: 0.62 to 3.39, p = 0.004)”. Only three studies were analyzed for the chronic effects of switching from combustible smoking to vaping e-cigarettes on heart rate, diastolic and systolic blood pressure. No effect was observed for heart rate. “On the other hand, electronic cigarette use significantly reduced both systolic blood pressure (pooled weighted MD = –7.00, 95% CI: -9.63 to -4.37, p <0.0001) and diastolic blood pressure (pooled weighted MD = –3.65, 95% CI: -5.71 to -1.59, p = 0.001)” ( Skotsimara *et al*., 2019).

Dollerup *et al.* found no significant differences in the time to diagnosis of ischemic heart disease or cerebrovascular disease in smokers who quit using NRT *vs.* no NRT at four weeks. However, after a year, there was increased diagnoses in participants using NRT ( Dollerup *et al*., 2017). Unfortunately, this study had several limitations, which may affect the conclusions. There was no information on any other diseases the participants may have been suffering from, and therefore, the doctors prescribing NRT may have been biased towards patients who were the sickest. In addition, there were no data provided on the utilization of the NRT, only prescription records, meaning that the NRT may have not been used as intended (or at all). Not only this, but patients may have supplemented the prescription with available over-the-counter drugs or continued to smoke. No information was collected on the amount each patient was smoking before their cessation attempt, or whether they were successful. No family history was recorded, which would have alerted the authors to a predisposition to cardiovascular disease in certain patients. A meta-analysis, however, also showed that NRTs were associated with a significant increase in cardiovascular events. When these events were examined further, the authors found the association was limited to less serious events (palpitations, bradycardia and arrhythmia). There was no increased risk in major adverse cardiovascular events (cardiovascular death, nonfatal myocardial infarction, and nonfatal stroke) ( Mills *et al*., 2014). An updated review comparing the risk of heart disease and stroke in users of either Swedish snus or American smokeless tobacco products showed there was no increase in risk found in Swedish snus users; however, there was an increase in risk of both heart disease and stroke in American smokeless tobacco product users. The authors speculate that this may be because of different levels of constituents present in the different products, but as nicotine is present in both products, it is unlikely to be the cause of the diseases observed. ( Rostron *et al*., 2018).

Arefalk *et al.* investigated whether there was an association between snus use and heart failure in two Swedish cohorts. A positive association was found but unfortunately, there were not enough exclusive snus users in one of the cohorts to carry out the analysis, and the authors included participants who had also smoked. The authors stated that residual confounding in this cohort could not be excluded. In the second, younger cohort, however, exclusive snus users could be identified and analyzed. A “borderline” significant association was found between snus use and heart failure, although no dose response relationship was shown. In this second cohort, no data was available for other potential confounding factors, such as alcohol consumption and diabetes ( Arefalk *et al.*, 2012). In an additional study by the same authors, snus cessation was associated with a significant reduction in death post myocardial infarction. The authors hypothesize several mechanisms by which this effect may manifest, such as arrhythmogenesis in response to nicotine, but only animal studies are available to support this theory. In addition, this cohort was also confounded by smoking history, as the number of exclusive snus users was too few to analyze in isolation. Alcohol use data was also not available for the participants of this study ( Arefalk *et al.*, 2014).

In a study comparing the immediate effect of nicotine on cardiovascular parameters, transdermal nicotine patches, nicotine nasal spray and cigarette smoking were all found to increase systolic and diastolic blood pressures as well as heart rate. Cigarette smoking produced the greatest increases, followed by the nasal spray and then patches. These results indicated that the mode of nicotine delivery also has an effect on cardiovascular outcomes ( Benowitz *et al*., 2002). In another study, no significant difference was observed in heart rate, diastolic blood pressure or systolic blood pressure in participants treated with 0, 21, 42 or 63 mg of transdermal nicotine. It should be noted that the participants were allowed to continue smoking, and so nicotine response may have already been maximal prior to patch application. If this was the case, treatment with further nicotine may not be expected to have additional effects ( Zevin *et al*., 1998). In support of these findings, however, smokers who had suffered a major coronary event and were treated with transdermal nicotine patches showed no increased risk of suffering adverse events during a 10 week period, compared to placebo patch treatment ( Joseph *et al*., 1996). The authors strongly concluded: “Concern about the use of nicotine replacement therapy for smokers with cardiovascular disease is not warranted” ( Joseph *et al*., 1996). Woolf and colleagues carried out a retrospective study in patients admitted to hospital for acute cardiovascular incidences and who were discharged with a prescription for NRT. They found that NRT use was not associated with an increased risk of adverse cardiovascular events after 1 year ( Woolf *et al*., 2012). Benowitz *et al.* also recently found no significant differences in the number of cardiovascular events in a group of 2022 healthy smokers who received NRT in the form of a patch, versus placebo ( Benowitz *et al*., 2018). It should be noted that participants with pre-existing cardiovascular disorders were excluded from the study. To investigate the effect of NRT in patients with coronary heart disease, a retrospective study was carried out on 4885 smokers who were hospitalized after myocardial infarction and received NRT within two days of admission. The participants were followed until hospital discharge and monitored for readmission within a month. The authors state “[T]he use of NRT products starting during the first 2 days of hospitalization was not associated with any significant differences (harm or benefit) in outcomes among smokers hospitalized with acute coronary heart disease” ( Pack *et al*., 2018). This further supports the evidence that there is little risk of nicotine use in the context of NRT. An ongoing clinical trial submitted in 2017 is looking to assess the cardiovascular effects of nicotine delivered in e-cigarettes over the medium term (6 months), and how this may affect use as a cessation aid ( Klonizakis *et al*., 2017).

In contrast, a study using sublingual nicotine delivery in tablet form (4 mg) to healthy smoking participants showed that heart rate, systolic and diastolic blood pressure, rate-pressure product (heart rate*systolic blood pressure), and sympathetic nerve activity to muscle circulation (MSNA) were all significantly increased in response to nicotine compared to a placebo control. Nicotine administration showed no effect on any of the breathing parameters measured (ventilation, tidal volume, minimal oxygen saturation etc.). The authors advised that the sympathetic nerve stress caused by nicotine should be considered when prescribing the use of NRT in smokers with limited coronary reserve and those at risk of sudden hypoxic events ( Najem *et al*., 2006). Similarly, in another study using sublingual administration of nicotine in healthy male non-smokers, administration of nicotine increased heart rate, systolic blood pressure, augmentation index (a marker of peripheral resistance) ( Mitchell *et al*., 2005) and carotid-femoral pulse wave velocity (a marker of arterial stiffness) ( Adamopoulos *et al*., 2009). The authors suggested that the changes in these measures were driven by an acute sympathetic response, as they were only recorded for one hour after administration. They suggest the chronic response may be different, because of the desensitization of nicotinic receptors known to occur over repeat nicotine exposures ( Adamopoulos *et al*., 2009). This hypothesis is supported by a study carried out in healthy female smokers. Arterial stiffness was found to increase after smoking a conventional cigarette; however, no change was observed after smoking an e-cigarette. ( SzołtySek-Bołdys *et al*., 2014). When participants were administered an acute exposure to Swedish snus, again, heart rate, diastolic and systolic blood pressure increased; however, when the data was interrogated further, it revealed that the female participants were driving this difference. There was no increase observed in the male participants. There was also no change in measures of arterial stiffness in either gender. The authors suggest that gender differences in hemodynamic responses may be responsible for this surprising result ( Antoniewicz *et al*., 2018). A gender effect was also reported when participants were administered nicotine intravenously. Acute heart rate response at 1- and 2-minutes post infusion was significantly increased in female participants compared to their male counterparts. It is unclear whether this difference is associated with any long-term clinical outcomes ( Jensen *et al*., 2018).

A study in Parkinson’s disease patients recently found that symptoms of low blood pressure, which are common and debilitating symptoms of the disease, could be effectively treated with nicotine gum. The participants, who were all non-smokers, chewed 4 mg gum over a period of 30 minutes, which elevated both diastolic and systolic blood pressure after 10 minutes, and was sustained for 90 minutes. No serious adverse events were observed and none of the patients reached hypertensive levels, leading the authors to propose nicotine gum as a potential treatment for low blood pressure in Parkinson’s disease patients; however, their sample size consisted of only 10 individuals, and therefore, further studies are needed to confirm these findings. Studies will also need to be carried out over longer periods, to observe the development of any resistance to the effects of nicotine ( DiFrancisco-Donoghue *et al*., 2019).

Although there are immediate pharmacological effects of nicotine on cardiovascular parameters, there is not enough evidence to suggest that there is an increase in risk to long-term cardiovascular health as a result of nicotine exposure from either NRT or e-cigarettes. More importantly, e-cigarette use is substantially more favorable when compared to conventional cigarette smoking. It is important to consider, however, that there is a lack of studies in participants who have been exposed exclusively to nicotine long-term (equivalent to decades of smoking). Overall, current studies indicate that the nicotine delivered by e-cigarettes does not increase the risk of cardiovascular events in individuals who do not have any underlying cardiovascular disease.

## Thrombosis, inflammation and atherosclerosis

### Human studies

A study in 20 non-smokers showed that markers of platelet activation were increased after vaping an e-cigarette, however, the authors acknowledged “We do not know if a single constituent of the liquid mixture or the constituents as a whole could be responsible for the observed changes in platelet function” ( Nocella *et al*., 2018). This observation was poignant in light of *in vitro* data, which showed that platelet activation was stimulated by e-liquid and e-liquid vapor, but suppressed by pure nicotine ( Hom *et al*., 2016).

A systematic review of three papers investigating the risk of stroke in human patients using NRT found no adverse effect; however, there was insufficient evidence to draw a definitive conclusion ( Lee & Fariss, 2017). Hergens *et al.* found no relationship between the risk of stroke and snus use in the cohort of Swedish construction workers. After the data was interrogated further, there was an increased risk of fatal ischemic stroke ( Hergens *et al.*, 2008); however, when this study was included in a meta-analysis, no overall effect was found ( Lee, 2011). In support of these findings, a study that pooled eight Swedish cohorts of male snus users also found no relationship with risk of stroke ( Hansson *et al*., 2014).

Endothelial progenitor cells (EPC) may be released from the bone marrow in response to endothelial injury. A study on 16 healthy, non-daily smokers showed that the number of EPCs was significantly increased in the blood of the participants after 10 puffs on an e-cigarette device. Levels returned to baseline within 24 hours after puffing. The authors state that this “[M]ay indicate an impact on vascular integrity leading to future atherosclerosis. However, further research is needed to understand the long-term effects of ECV” ( Antoniewicz *et al*., 2016).

Since the submission of this paper, two new studies have been published that the reviewers felt important to include. Kuntic *et al.* found a significant reduction in markers of endothelial function 15 minutes after healthy smokers vaped an e-cigarette. The authors followed-up their observations in humans by investigating the effects of vascular function in mice. When mice were exposed to e-cigarette vapor for 20 minutes, 6 times a day over three days, impairment of endothelial function was also observed; however, the only consistently significant changes were seen in mice exposed to e-vapor without nicotine. The human participants were exposed to nicotine-containing e-liquid only so it is impossible to say whether the effects seen would have been more profound if non-nicotine e-liquid was also tested, and therefore, it is difficult to isolate an effect of nicotine alone. Further, *in vitro* experiments in cultured human endothelial cells highlighted the role of oxidative stress as a possible mechanistic pathway involved in the manifestation of the endothelial dysfunction observed. The human participants in the study were all smokers. It is also possible, therefore, that their previous smoking history had already led to compromised endothelial function. Chronic effects of e-cigarette vaping on endothelial function cannot be extrapolated from any of the results in this study ( Kuntic *et al.*, 2019). In contrast to the findings of this research, a significant improvement in endothelial function was observed in healthy smokers who switched to e-cigarettes for 1 month. The improvement was similar for smokers who switched to e-liquids containing nicotine and nicotine-free e-liquids. The consistent observation across these studies is that it appears that nicotine may not be a primary factor in the deterioration of endothelial function. If this is the case, it will also be important for future research to isolate any potential ingredient in e-liquids that may cause endothelial dysfunction ( George *et al.*, 2019).

Nicotine has been reported to have both pro- and anti-inflammatory effects ( Benowitz & Burbank, 2016). In human studies involving the use of NRTs in smoking cessation, participants have shown a reduction in inflammatory markers. It is thought that stimulation of the α7-nicotinic acetylcholine receptor (nAChR), on the macrophage cell surface leads to reduction of pro-inflammatory cytokine production, preventing sepsis ( Lee & Cooke, 2011). Contrastingly, sympathetic nervous stimulation by nicotine could also induce an inflammatory response by stimulating monocyte production ( Benowitz & Burbank, 2016).

### Animal studies

In an atherosclerotic mouse model (ApoE deficient), nicotine was found to enhance atherogenesis through its angiogenic action; however, it did not initiate plaque formation, only enhanced the growth of existing plaques ( Heeschen *et al*., 2001). Another study confirmed that short-term exposure to nicotine in mice also led to increased angiogenesis, however, chronic exposure abolished this effect. When mice were exposed to nicotine for 52 weeks, there was a 33% reduction in the number of vascular sprouts ( Konishi *et al*., 2010). It is clear that vascular response to nicotine exposure is complex, and in human models, short-term studies may not reflect the ‘real-life’ responses to nicotine over years or even decades of intake. Differences in response to nicotine over time were also observed in thrombus formation in mice. C57BL/6 J mice were treated with 100 μg/ml in their drinking water for eight weeks (chronic exposure) and compared to an acute intravenous treatment of 3 mg/kg. No effects were observed on thrombus formation or platelet aggregation in the chronically exposed mice, however, the acute exposures caused a significant reduction in arteriole occlusion time in female mice only ( Lindenblatt *et al*., 2007). Not only does this study add to the evidence for differences between acute and chronic exposures, but it also adds sex as a further confounding factor. Additionally, a study that chronically exposed female rats to nicotine vapor (20 hours a day, five days a week for up to two years) and produced plasma nicotine concentrations equivalent to a heavy human smoker, showed no difference in myocardial pathologies and no atherosclerotic lesions when compared to controls ( Waldum *et al*., 1996).

Fahim *et al.* found that white blood cell, red blood cell and platelet counts, as well as hemoglobin, and hematocrit did not change when mice were treated with intraperitoneal (i.p.) nicotine (1 mg/kg) for three weeks. Despite this, pial cerebral microvessel thrombosis was significantly increased in both arterioles and venules, suggesting a higher susceptibility to cerebral thrombosis (and potentially stroke) in response to nicotine ( Fahim *et al*., 2014).

In rats treated intraperitoneally with nicotine for 28 days, aortic remodeling was observed as well as a loss of aortic reactivity. The authors measured markers of oxidative stress and found an increase, which they hypothesized could be the mechanism stimulating the aortic changes in response to nicotine. The nicotine-exposed rats showed increased blood pressure during the experiment, despite the dosage of nicotine being equivalent to “light smoking”, and not sufficient to cause any changes in body weight ( Zainalabidin *et al.*, 2014). It is unclear whether these observations may translate into a human model as there have been no studies to characterize the effect of long-term nicotine exposure on blood pressure parameters without smoking as a confounding factor.

Nicotine has also shown chemotactic properties, leading to accumulation of neutrophils to the endothelium ( Filippini *et al*., 2012). Rats exposed to saline vapor containing 5% nicotine showed an increase in white blood cell count for up to 24 hours post exposure compared to saline controls. Interestingly, 10% nicotine exposure only resulted in smaller increases in white blood cell count at 6 hours post exposure, suggesting the response did not increase with dose ( Ahmad *et al*., 2019).

Clinical observations have suggested a protective effect of smoking on the inflammatory disease, ulcerative colitis. Nicotine has been proposed as the constituent in tobacco smoke to bring about this effect, and has shown clinical efficacy ( Pullan *et al.*, 1994). Studies in mouse models of ulcerative colitis have supported this hypothesis, showing decreased activation of inflammation through both miR-124/STAT3 ( Qin *et al.*, 2017) and MAdCAM‐1 ( Maruta *et al.*, 2018) pathways.

### *In vitro* studies

In addition to sympathetic stimulation by nicotine through nAChR leading to vasoconstriction, nAChR are also expressed on endothelial cells (among other cell types). *In vitro* studies of endothelial cells have shown that nicotine stimulates proliferation ( Villablanca, 1998), increases the expression of nitric oxide synthase ( Zhang *et al*., 2001), and alters the production of both fibroblast growth factors and matrix metalloproteinases ( Carty *et al*., 1996). These studies offer mechanisms by which nicotine could play a role in the development of pathological angiogenesis, atherosclerosis and platelet aggregation.

EPC numbers increased when isolated from mice exposed to nicotine for one month; however, after three and six months of exposure, EPC cell numbers were reduced compared to non-exposed controls. Further *in vitro* assays suggested that EPCs from mice exposed to nicotine for six months demonstrated decreased proliferation, telomerase activity, nAChR epression and SIRT1 expression ( Li *et al.*, 2017). The results suggest that EPC response to nicotine short-term may differ from the long-term effects, and therefore acute studies in humans may not be representative of longer-term health effects.

The relationship between nicotine exposure and thrombosis, atherosclerosis and inflammation are yet to be conclusively defined. Current evidence suggests that there is little risk of stroke associated with nicotine intake in humans, however, specific subtypes or severities of stroke may show a different relationship. It is clear from in vitro and animal studies that nicotine can elicit a response on endothelial function. Acute response to nicotine in humans suggests that this may also translate to EPCs in vivo. It is likely, however that the relationship is complex, with evidence suggesting that the effects of acute exposure differs markedly from chronic. It is also unclear what clinical manifestations may arise as a result. Nicotine appears to offer anti-inflammatory properties against certain chronic diseases such as ulcerative colitis, but given its potential to show pro-inflammatory stimulation, extensive study into the systemic effects is necessary.

## Carcinogenesis

Several studies have proposed mechanisms by which nicotine may activate molecular pathways leading to the propagation of tumorigenesis *in vitro*. Studies involving nicotine exposures in the whole organism, however, have repeatedly failed to show a significant relationship between nicotine and cancer initiation. Several documents issued by authoritative bodies also state that nicotine is not considered a carcinogen. A summary of these can be found in Table 1.

**Table 1.** Summary of official statements surrounding the carcinogenic properties of nicotine.

Authority	Statements
Surgeon General ^a^	“…findings of animal studies do not support the hypothesis that nicotine is a complete carcinogen”. “There is insufficient data to conclude that nicotine causes or contributes to cancer in humans”.
Food and Drug Administration ^b^	Listed as a “Reproductive or Developmental Toxicant” and “Addictive” but not as a carcinogen.
Royal College of Physicians ^c^	“Studies carried out in experimental animals largely indicate that nicotine alone is not carcinogenic. *In vitro* and *in vivo* studies in animals do, however, suggest that nicotine can have tumour-promoting effects through activation of intracellular signalling pathways”.
National Academies of Science, Engineering and Medicine ^d^	“Current evidence does not support the idea that nicotine is a human carcinogen, let alone a complete carcinogen”. “Based on the existing body of evidence, it is reasonable to infer there is likely no significant increase in risk of cancer from exposure to nicotine delivered by e-cigarettes”.

^a^ The Health Consequences of Smoking—50 Years of Progress: A Report of the Surgeon General ( Surgeon General, 2014)

^b^ Harmful and Potentially Harmful Constituents in Tobacco Products and Tobacco Smoke: Established List

^c^ Nicotine without smoke: Tobacco harm reduction. A report by the Tobacco Advisory Group of the Royal College of Physicians

^d^ National Academies of Sciences, Engineering, and Medicine. (2018). Public health consequences of e-cigarettes. Washington (DC), The National Academies Press

### Human studies

Analysis of data from the Lung Health Study assessed whether NRTs could contribute to cancer development ( Murray *et al*., 2009). During the study, 3923 participants were randomized to receive smoking intervention, and followed over a 5-year period. Cox regression models were used to predict the contribution of NRT (nicotine gum) to the incidence of cancer occurrence observed in the participants after a 7.5-year follow-up. The use of NRT was not associated with the incidence of cancer in any of the prediction models used. There were, however, considerable limitations to the study design; for example, all the participants were previous smokers. It is impossible, therefore, to say whether any of the cancer incidences observed were because of nicotine gum or previous exposure to carcinogens from cigarettes. It could be that the effect of smoking on cancer is so strong that it eclipsed a potentially small effect from nicotine gum. In addition, the participants had been using nicotine gum for the 5-year duration of the study but had been smoking for much longer prior to that time (taken from the mean number of pack years at baseline). It may be that 5 years was not a long enough exposure to compare to the risk from smoking ( Murray *et al*., 2009) and that the overall length of the study may not be enough time to observe manifestation of lung cancer at all. This is supported by the overall low numbers of lung cancer incidence in the cohort (n = 75).

A retrospective study was carried out in a cohort of 279,897 snus-using Swedish men, followed from 1978–1992. No increased risk was observed for either oral or lung cancer in non-smokers who used snus. There was, however, an increased risk of pancreatic cancer associated with snus use, and this was positively associated with dose (relative risk (RR): 1.9 (0.8-4.3) at 1–9 g/day; 2.1 (1.1-3.8) at ≥10 g/day) ( Luo *et al*., 2007), although major risk factors for pancreatic cancer (alcohol use and diabetes) were not adjusted for in this study ( Nilsson, 2017). It must be noted that snus it not a source of nicotine alone and contains other carcinogenic products from tobacco. Specifically, these include 4-(nitrosomethylamino)-1-(3-pyridyl)- 1-butanone (NNK), which has been shown to cause pancreatic cancer in a rat model ( Rivenson *et al*., 1988). Therefore, NNK, for example, could have been the primary carcinogenic factor in this cohort of men, rather than nicotine. Conversely, in a pooled analysis of nine cohorts of men across Sweden, totaling 418,448 participants from 1978 to 2013, there was no increase in the incidence of pancreatic cancer found in snus users ( Araghi *et al*., 2017). The authors concluded, “Our findings, from the largest sample to date, do not support a role of snus use in the development of pancreatic cancer in men. They, furthermore, point to tobacco smoke constituents other than nicotine or its metabolites, *i.e.*, carcinogens associated with combustion, as the causal agent explaining the increased risk of pancreatic cancer in smokers”. Araghi *et al.* adjusted for both alcohol consumption and type 2 diabetes, suggesting that the observations by Luo *et al.* may have been due to confounding. The cohorts used in the Araghi *et al.* study also spanned a more recent time period. The tobacco-specific nitrosamine content of smokeless tobacco in Sweden has decreased during this time ( Osterdahl *et al*., 2004), which would have also decreased smokeless tobacco user exposure to these carcinogens.

### Animal studies

A thorough review by Haussmann and Farriss, which analyzed studies investigating carcinogenic effect of nicotine in animal models, concluded that there was no evidence to suggest that nicotine is a complete carcinogen ( *i.e.*, causes tumor initiation, promotion and progression). When analyzing studies assessing whether nicotine could modulate carcinogenesis, there was significant variation in results. This may be because of differences in study design, or the animal models used (e.g., immunocompromised *vs.* competent, dose, delivery method etc.). The authors concluded that there is enough evidence to state: “[N]icotine can stimulate carcinogenesis of inoculated cancer cells in laboratory animals, especially immunocompromised mouse models”. There is insufficient evidence to demonstrate whether nicotine could modulate chemically induced cancer ( Haussmann & Fariss, 2016). The overwhelming message of this review was that the data currently available is not sufficient to draw definitive conclusions. Well-designed and implemented studies, particularly in humans, are required to address the uncertainty once and for all.

### *In vitro* studies

The role of nicotine in cancer modulation *in vivo* has been supported by several *in vitro* studies, which elucidate nicotine-stimulated pathways leading to cellular proliferation, angiogenesis and resistance to apoptosis. A transcriptome sequencing study showed that exposing normal breast epithelial cells (MCF-10 A) to nicotine altered the expression of 138 transcripts by more than twofold. Upstream analysis and gene ontology isolated matrix metalloproteinase 2 (MMP2) as a potential candidate for promoting tumor progression after nicotine exposure ( Bavarva *et al*., 2013). MMP2 is an enzyme that cleaves molecules involved in signal transduction, and so may have many roles *in vivo*. This study was preliminary, providing an unbiased approach to detecting changes to transcription. Analysis at the RNA level, however, is limited because levels of transcription are not always reflected by levels of translation and action at the protein level. Although this study provides a good list of candidates for future investigation, it is impossible to deduce what mechanisms are involved from the results thus far.

A study by Nishioka *et al.* showed that nicotine exposure leads to the proliferation of cells that are already DNA damaged, through the overriding of the G1/S checkpoint. Human and lung epithelial cell lines were exposed to nicotine (0.5 µM) after damage had been induced by either gamma radiation or benzopyrene. Although cell proliferation was not restored to the level seen in undamaged cells, there was a significant increase in the levels of proliferation observed with the addition of nicotine, compared to the damaged cells alone. Corresponding increases in cyclins A and D1 were observed with the nicotine treatment. Checkpoint kinase 2 (Chk2) protein is phosphorylated in damaged cells leading to activation, cell cycle arrest, DNA repair or apoptosis. When damaged cells were treated with nicotine, Chk2 remained unphosphorylated. Interestingly, there seemed to be no effect of nicotine on the G2/M phase. This study shows a novel mechanism by which nicotine may act to promote the survival and proliferation of damaged cells. The experiments shown were carried out *in vitro* and therefore caution must be taken when extrapolating to the whole organism. If applicable in the human model, however, this mechanism could present a role for nicotine in compounding the carcinogenic effects of other tobacco smoke components. ( Nishioka *et al*., 2011). The Surgeon General summarized this point in its 2014 report, “[T]here is substantial experimental evidence indicating that nicotine is bioactive for a number of carcinogenic mechanisms in experimental systems. Although *in vitro* data are suggestive of relevant biological activity, this is not supported overall by the most recent experimental animal studies” ( Surgeon General, 2014).

## Metastasis

### *In vitro* studies

**Figure 2.**
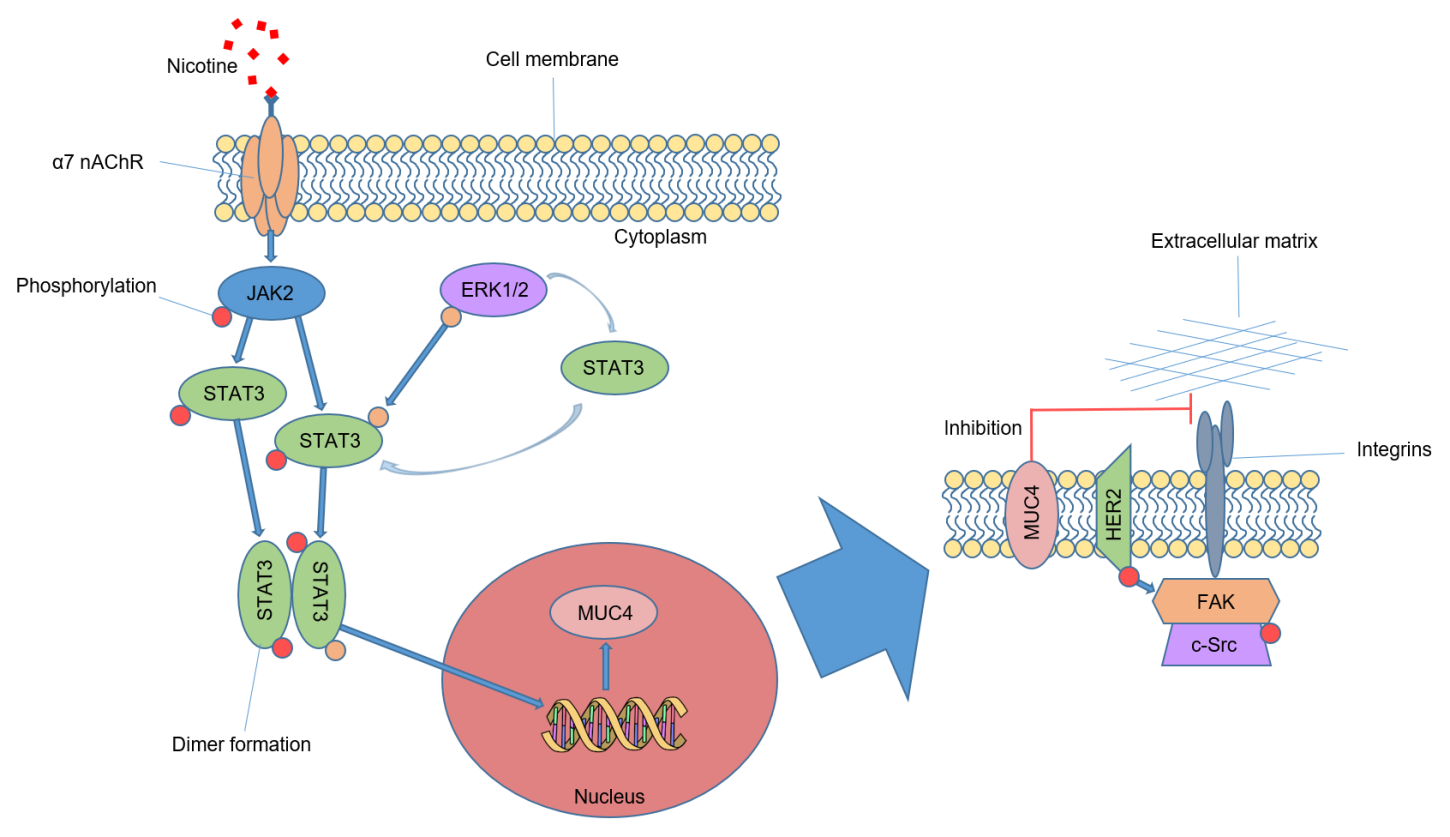
Nicotine induced stimulation of pancreatic cancer cell metastasis *in vitro*. Nicotine interacts with the α7 nAChR on the cell surface, activating JAK2 and leading to the phosphorylation of STAT3. ERK1/2 mediated STAT3 phosphorylation is known to enhance DNA binding and to stabilize STAT3 in the cytoplasm. The phosphorylated forms of STAT3 join to form dimers and translocate into the nucleus where they interact with the DNA causing upregulation of MUC4. MUC4 is then expressed on the cell membrane and either directly inhibits the integration of integrins with extracellular matrix, or indirectly through HER2 activation. These pathways lead to a decrease in cell-to-cell adhesion and increase the migratory potential of pancreatic cancer cells. *This figure has been reproduced with permission from Momi et al., 2013*.

There is very limited data surrounding the role of nicotine exposure to metastasis; however, a possible effect has been found in cell lines exposed to nicotine. To investigate the increased occurrence of pancreatic cancer in smokers, a range of pancreatic cancer cell lines were used (CD18/HPAF, Capan1 and FG/Colo357) as well as non-cancerous human pancreatic ductal epithelial cells and exposed to a range of nicotine concentrations (0.1 µM, 1 µM and 5 µM). A rise in the α7 subunit of α7 nAChR was observed along with a corresponding dose-dependent upregulation of mucin 4 (MUC4) expression at both the protein and RNA levels. The increase in MUC4 could be attenuated using an α7 nAchR antagonist and led to increased migration of cells in a wound healing assay. Further downstream analysis led to the proposal of the pathway outlined in Figure 2 ( Momi *et al*., 2013). The authors confirmed their observations in a mouse model; unfortunately, they used a cigarette smoke exposure, so the effect cannot be attributed specifically to nicotine. This study is preliminary in terms of design and cannot be extrapolated into a human model.

Supporting the findings of this study, an *in vitro* model of head and neck cell carcinoma showed that nicotine at a concentration of 0.5 µM increased cell proliferation and viability, as well as metastatic characteristics through activation of the epidermal growth factor pathway ( Shimizu *et al*., 2019). Only one concentration of nicotine was used, which was approximately three times higher than that achieved in the blood plasma of humans using NRT or e-cigarette devices ( Kyte & Gewirtz, 2018). Although these experiments demonstrate potential pathways of nicotine action, *in vivo* data is required to establish whether they are relevant to the cancer in the organism.

## Reproductive effects

### Male fertility

Male fertility is more sensitive to the effects of nicotine compared to female fertility ( Jorsaraei *et al*., 2012). Nicotine concentrations of between 70 and 300 µg/L (0.43–1.85 µM) have been found in the seminal fluids of daily smokers ( Oyeyipo *et al*., 2014), suggesting high transfer from the blood stream, and therefore, testicular cells and spermatogenesis may be vulnerable to its effects.

**Figure 3.**
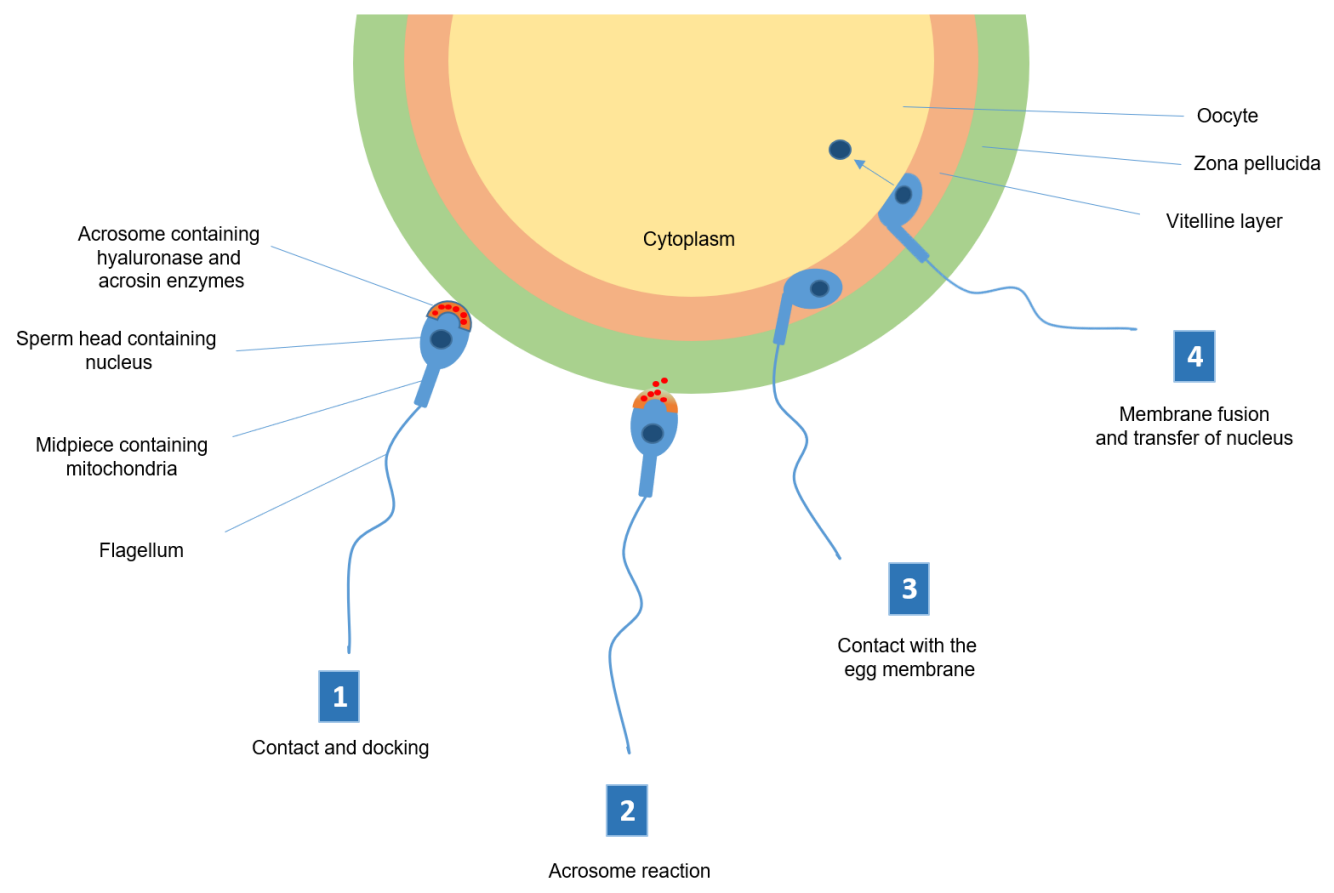
Schematic diagram of the acrosome reaction and fertilization. 1) Sperm cells are chemo-attracted to the oocyte and once in close proximity, interact with zona pellucida (ZP) glycoproteins. The arrangement of these glycoproteins are species specific and are responsible for preventing cross species fertilization. 2) ZP glycoproteins initiate a signaling cascade through receptor binding on the sperm surface leading to a degradation of the acrosome membrane and release of hyaluronase and acrosin, which breaks down the ZP. 3) Once through the ZP, the sperm enters the vitelline space and contacts the oocyte membrane. Binding occurs at the posterior portion of the sperm head. 4) The membranes of oocyte and sperm fuse, causing a rapid depolarization and hardening of the ZP, preventing polyspermy. The nucleus of the sperm enters the oocyte and the fertilized oocyte completes second meiosis.

The primary function of sperm cells is to fertilize the oocyte, a task that is reliant on viability, motility and a successful acrosome reaction. The acrosome is an enzyme-filled capsule on the head of the sperm, which reacts to break down the zona pellucida of the oocyte on contact ( Figure 3).

### Animal studies

*In vivo* studies have mostly been carried out in rat models. Daily administration of oral nicotine at 0.5 and 1.0 mg/kg body weight (considered in the range of human exposure through cigarette smoking) ( Jana *et al*., 2010) for four weeks was found to significantly reduce the count and motility of sperm in rats in a dose-dependent manner, although there was no change in viability ( Oyeyipo *et al*., 2011). The reduction in motility could have been because of a significant increase in the occurrence of spermatozoa with “curve tail”, a change in morphology resulting in a U shape forming in the tail. In addition to the sperm parameters tested, the rats from this study also showed a decrease in libido, shown by a reduction in mounting attempts. None of the untreated female rats housed with males from the high dose treatment group conceived during the study period, compared to 100% conception in the untreated male group. This effect was reversed after nicotine was withdrawn ( Oyeyipo *et al*., 2011).

Consistent with the observations above, daily i.p. injection of nicotine at 0.6 mg/kg body weight in rats for six weeks led to seminiferous tubule and spermatogenic derangement in the testes as well as reduced overall testicular weight ( Jana *et al*., 2010), also observed in orally administered rats ( Oyeyipo *et al*., 2010). These effects could be partially rescued by the human chorionic gonadotropin (stimulating androgenesis) or taurine (an antioxidant) co-supplementation ( Jana *et al*., 2010). There was a reduction in the number of germ cells at several generations in the sperm cycle and abnormalities to sperm morphology were also significantly increased, particularly in the sperm head and formation of a “banana-like” shape. Not only were plasma testosterone levels decreased after nicotine administration, suggesting impaired androgenesis, but also luteinizing hormone (LH) and follicle stimulation hormone (FSH) levels, indicating disruption of normal hormonal release from the pituitary gland. The authors hypothesized that most of the effects shown in sperm were a result of the reduction in testosterone, caused by nicotine decreasing LH and FSH plasma concentrations (potentially by increasing glucocorticoid release from the adrenal gland), and inhibiting the production and activity of androgenic enzymes. Other contributing factors may include DNA damage and an increase in reactive oxygen species ( Jana *et al*., 2010). Very similar findings have been shown in a recent study in adolescent rats, which were administered 0.6 mg/kg of nicotine via i.p. injection over 12 weeks ( Budin *et al*., 2017). A higher concentration of subcutaneously injected nicotine in rats (2.5 mg/kg body weight) also resulted in smaller testes and smaller seminiferous tubule diameter, as well as reduced serum testosterone compared to untreated controls ( Abd El-Aziz *et al*., 2016).

### *Ex vivo* studies

*Ex vivo* exposure of sperm from healthy, non-smoking men to a range of nicotine concentrations over three hours was found to decrease both the number of viable sperm and their motility. There was also an increase in the number of cells that had undergone a spontaneous acrosome reaction, disabling their ability to fertilize the oocyte. The negative effects of nicotine, however, were only found when sperm were exposed to concentrations far beyond physiological (1–10 mM). The sperm were also washed of all seminal fluids prior to exposure, so the effects seen cannot be applied to the biological environment ( Oyeyipo *et al*., 2014). A similar experiment looked at the kinetic parameters of sperm from 10 healthy men exposed to nicotine and cotinine at multiple concentrations *ex vivo*. The dose that corresponded to the physiological exposure in smokers (70 ng/ml) had no effect on the kinetic parameters. There was a reduction in the kinetic properties of sperm only when a high dose (35 µg/ml) of nicotine was applied ( Jorsaraei *et al*., 2012).

Results from the studies above seem to imply a disparity between the *in vivo* and *ex vivo* effects of nicotine on male fertility. There are several reasons why this may be the case. The male humans who participated in the *ex vivo* studies were all non-smokers. It may be that by the time the sperm is mature and ejaculated, the cells are not as vulnerable to the effects of nicotine. They would have also had a ‘normal’ physiological environment during spermatogenesis. Not only this, but the period of exposure was much shorter (hours) compared to the *in vivo* models (weeks), which may not be enough time to observe an effect. In the *in vivo* models, the animals were exposed to nicotine for longer periods, which corresponded to multiple stages of spermatogenesis and are more reminiscent of the longer-term usage seen in ‘real-life’ smokers. In these cases, the disruption to androgenesis seemed to be key in the sub-fertility observed. We cannot, however, overlook the possibility of species differences between human and rodent models. There are no studies that investigate fertility in men exposed exclusively to nicotine. This includes a lack of observational studies in men using NRTs. Although cigarette smoking introduces considerable confounders compared to nicotine exposure alone, many studies report a negative effect of smoking on both sperm parameters ( El Mulla *et al*., 1995; Evans *et al*., 1981; Shi *et al*., 2001; Vine *et al*., 1996) and *in vitro* fertilization outcomes ( Joesbury *et al*., 1998; Zitzmann *et al*., 2003). Given the similarities between the observations in male human smokers and the nicotine-exposed rats, it may be plausible that at least some of the characteristics shown in men may be as a result of nicotine exposure. Further studies are needed to clarify this relationship.

## Female fertility

The female reproductive system has been reported to be less sensitive to nicotine compared to the male ( Riesenfeld & Oliva, 1987). Women metabolize nicotine and cotinine (the principle metabolite of nicotine) much faster compared to men ( Benowitz *et al*., 2006). This increased metabolism is an estrogenic effect, and could offer a protective mechanism; however, the hypothesis that nicotine is associated with reduced fertility in females remains. Unfortunately, there are no studies into the effects of nicotine on fertility in women, although cotinine can be detected in the follicular fluid of women exposed to cigarette smoke ( Zenzes *et al*., 1996), showing plausibility for an effect directly on the reproductive cells.

### Animal studies

A study in mice showed that subcutaneous injection of nicotine (5 mg/kg/day) for 30 days resulted in a significant increase in the number of apoptotic oocytes after superovulation. This effect could be rescued with co-administration of vitamin E (60 mg/kg/day orally) ( Asadi *et al*., 2013). Vitamin E acts as an antioxidant, and therefore its effect on oocyte viability suggests that nicotine may cause an increase in oxidative stress. The dosing regimen used in this study is unclear. One would assume that the entire dose was not administered in one injection, as the lethal dose of nicotine in mice is reported to be 3.3 mg/kg ( Mayer, 2014). There was no justification for the dosage used.

Similarly, i.p. injection of nicotine at 6.25 ng/g body weight in rats either twice, three or four times a day, showed a dose-dependent reduction in the number of oocytes ovulated. When animals were treated with cotinine in the same regimen, no difference was found, suggesting a nicotine-specific mechanism. The effect on ovulation was accompanied by a reduction in circulating estrogen concentrations ( Blackburn *et al*., 1994).

Bordel *et al.* hypothesized that follicular vascularization would be altered by treatment with nicotine, because of studies suggesting the positive effect of nicotine on angiogenesis in tumorigenesis models. They transplanted follicles from donor hamsters into recipients with a dorsal skin-fold chamber, so that follicular vascularization could be observed over time in a live animal model. Angiogenesis was not changed in either of the nicotine treatment groups compared to controls, although capillary diameter was increased. Interestingly, however, follicles of the nicotine-treated animals showed higher levels of apoptosis in the granulosa cells surrounding the oocyte, and a subsequent decrease in follicular size ( Bordel *et al*., 2005). Granulosa cells interact closely with and support the maturing oocyte, so a reduction in granulosa cell number could lead to subnormal oocyte development.

In rhesus monkeys, injection of 0.05 mg/kg nicotine resulted in alterations to fallopian tube motility that was dependent on the stage of the menstrual cycle. This could result in alterations to oocyte transit from ovary to uterus – a critical point for conception. Unfortunately, the authors could not rule out a possible confounding effect of adrenaline, which was shown to increase as a result of stress during the experimental procedure, and may have also altered the contractile characteristics of the fallopian tube ( Neri & Marcus, 1972).

Rats treated with nicotine via transdermal patch showed no difference in the number of implanted fetuses or in integrin β3 staining of the endometrium, a marker of endometrial receptivity, compared to non-treated controls. The authors stated that the nicotine concentration achieved was equivalent to a 70 kg woman smoking 15 cigarettes per day, and were treated for three cycles before pregnancy was achieved. ( Akpak *et al*., 2017).

It is clear that the effect of nicotine on female fertility is far from fully understood. There is a lack of consistency between animal models, dosing regimen and hypotheses, and the results do not allow any clear conclusions for the reproductive outcomes in nicotine-exposed animals. Although there are many indications of possible cellular-level alterations in response to nicotine, there is no evidence to suggest a reduction in conception rates or number of offspring, which is the primary measure of fertility. It is impossible, therefore, to begin to formulate a hypothesis in humans. This is an area where further research would be beneficial. Currently there is not enough evidence to suggest that nicotine is detrimental to female fertility.

## Pregnancy and the offspring

There is a consensus that use of NRTs during pregnancy is less damaging to the fetus than smoking cigarettes, but should only be recommended if a pregnant mother is unlikely to stop smoking by any other means ( Dempsey & Benowitz, 2001). Nicotine exposure is considered as detrimental to the developing fetus, however, there is very little data that examines the effects of nicotine on human fetal growth without the confounding effects of cigarette smoking at various time-points during gestation. The FDA deem nicotine as a category D drug: “For pregnancy category D, if there is positive evidence of human fetal risk based on adverse reaction data from investigational or marketing experience or studies in humans, but the potential benefits from the use of the drug in pregnant women may be acceptable despite its potential risks” ( Food and Drug Administration, 2008). For this reason, there is a reluctance to commission studies involving the use of NRTs in pregnant women, due to fear of any detrimental consequences. Many women, who have taken part in studies that make use of NRTs during pregnancy, also smoke concurrently ( Oncken *et al*., 2008). There is also a high chance of misclassification because of the stigma surrounding smoking during pregnancy and the consequential likelihood of women misreporting their actual cigarette consumption ( Dietz *et al*., 2011).

Other confounding factors must also be considered when looking at nicotine intake during pregnancy. Many studies compare the risk of adverse outcomes between smoking and NRT, however, NRTs do not contain the other chemical constituents associated with cigarette smoking, and therefore risk reduction may be because of a reduction of other compounds. In addition, the nicotine itself is often delivered at much lower concentrations using NRTs compared to cigarette smoking ( Bruin *et al*., 2010), and therefore the direct effect of nicotine on pregnancy and fetal outcomes may be masked by changes in dose. This could be a key factor when considering the use of nicotine in e-cigarettes during pregnancy, as these products may be able to deliver comparable concentrations to cigarette smoking.

## Uterine blood flow

### Human studies

A study of 35 women investigated uterine and umbilical blood flow first after smoking a single cigarette, and then, approximately two weeks later, chewing a piece of nicotine gum. Both non-smoking mothers and habitual smoking mothers were included in the study and compared. The only statistically significant effects were seen in mothers who were habitual smokers; no significant effects of either smoking or chewing nicotine-containing gum was observed in non-smokers. In the habitual smoking group, there was an increase in the flow velocity waveform systolic-to-diastolic ratio of the umbilical artery after chewing the gum, and in those who smoked more than 10 cigarettes a day, a similar effect was observed after smoking the cigarette. The authors hypothesized that the effect could be a result of more efficient smoking and therefore nicotine uptake in the habitual smokers, as well as an increased number of nicotinic receptors. However, biological markers of nicotine uptake were not measured. No effect was seen on uterine blood flow in any of the groups. Although the authors cite differences in species and study design to explain these contradictory findings, the data looks to trend towards a positive correlation. It could be that small sample numbers and high variation are masking a potential outcome ( Bruner & Forouzan, 1991).

In a pilot study, six women who smoked during pregnancy were treated with a nicotine patch after 12 hours of smoking abstinence. Parameters of “fetal wellbeing” were then measured. Maternal salivary nicotine concentration increased as expected, but fetal heart rate, umbilical artery Doppler assessment and uterine contractility were not altered by the treatment. This is in contradiction to the uterine blood flow measurements found in the rat, although all the mothers in the study had been smokers throughout their pregnancies, and at treatment were already in their third trimester. Therefore, uterine blood flow may have been lower compared to non-smoking mothers. The dose of nicotine delivered was also much lower (21 mg) compared to the rat study, meaning that concentrations may be too low to produce a measurable outcome. The effects of nicotine patch treatment were only observed over a six-hour period and for one patch treatment. The effects of prolonged nicotine patch exposure were not tested ( Wright *et al*., 1997). This could also explain a difference in outcome with results from a study looking at fetal parameters during 24-hour nicotine patch application over four days. Fetal heart rate was found to be reduced in comparison to baseline (measured a week previously during normal maternal smoking behavior) in the mornings of the 2nd, 3rd and 4th days of testing. The reduction was not considered to be clinically significant for fetal outcome ( Ogburn *et al*., 1999). A strength of this study was that the participants were admitted to the study as inpatients, so their smoke-free status could be closely monitored; however, only 21 participants were included and the previous exposure of the fetuses to daily cigarette smoke may have affected the baseline measurements ( Ogburn *et al*., 1999).

### Animal studies

In pseudo pregnant rats injected with 0.5 mg/kg body weight, uterine blood flow was significantly reduced from 20–120 minutes post injection, without decreasing cardiac output. When injected with 5.0 mg/kg body weight, cardiac output and uterine blood flow were both significantly decreased. In the high dose group, uterine oxygen tension was also measured and was found to be significantly reduced. The low dose group was not measured for this parameter. The authors acknowledge that the concentrations of nicotine used in their experiment exceed the exposure obtained from smoking a cigarette, but suggest that low concentrations in the human may be enough to create a hypoxic fetal environment ( Hammer *et al*., 1981).

In a more clinically relevant model, pregnant rats were exposed to nicotine aerosol inhalation (NAI), which resulted in blood nicotine concentrations comparable to those after human smoking. The data showed that “NAI exposure in dose and kinetics equivalent to that in human smoking stimulates autonomic nAChRs resulting in disturbances in cardiac function and systemic hemodynamics as well as vasoconstriction of the uterine artery that disrupt uteroplacental hemodynamics, which can lead to fetal ischemia”. The authors pointed out that the placenta has a large reserve capacity, which may act as a buffer to the hemodynamic effects of nicotine exposure. It is therefore unknown whether the transient depression in uterine blood flow in response to smoking a cigarette may lead to an outcome for the fetus ( Shao *et al*., 2017).

## Birth outcomes

During pregnancy, the placenta, in addition to its role in transferring nutrients, gas and waste to and from the fetus, acts to protect against toxicological insult. In the case of nicotine, however, higher concentrations have been found in the fetal serum, placenta and amniotic fluid compared to maternal serum concentrations ( Luck *et al*., 1985). Although the placenta slows the uptake of nicotine to the fetus, it also slows its clearance, leading to an accumulation and higher exposure in the fetus compared to the mother ( Fischer *et al*., 2017; Slotkin, 2008). Therefore, even in isolation, away from the additional harmful effects of cigarette smoke, the direct effect of nicotine on fetal development should be considered. The mode of nicotine delivery has also been found to lead to different levels of accumulation in the fetus, with transdermal patches leading to the highest fetal exposures ( Slotkin, 2008). The authors did not examine the type of NRT used separately in this study.

### Human studies

Two studies used data from participants from the Danish National Birth Cohort to investigate the impact of nicotine on birth outcomes. Using NRT did not increase the risk of stillbirth when compared to stillbirths in non-smoking and non-NRT-using pregnant mothers, however, other safety outcomes (e.g., preterm birth, low birth weight etc.) were not measured ( Strandberg-Larsen *et al*., 2008). Morales-Suárez-Varela *et al.* investigated the incidence of congenital malformations in NRT-using pregnant mothers. NRT use in the first 12 weeks of pregnancy was associated with a relative prevalence rate ratio of 1.61 across all congenital malformations and 2.05 for musculoskeletal malformations. Interestingly, there was a greater prevalence of malformations in mothers who used NRT compared to smokers. The authors hypothesized that this may be due to a higher number of pregnancy losses in smokers, leading to fewer live births with congenital malformations. Data on miscarriages was not included in the analysis and the conclusions were based on 19 malformations in 250 pregnancies ( Morales-Suárez-Varela *et al*., 2006). A systematic review and meta-analysis of five randomized control trials of NRT use in pregnant women showed no significant differences in pregnancy outcomes (preterm birth, perinatal mortality, fetal deaths, neonatal intensive care admissions or miscarriage) for the women who received NRT, *vs.* those who did not. The authors point out that this could be as a result of non-adherence of the participants ( Coleman *et al*., 2011).

A recent study carried out in pregnant women from the UK found that NRT was not associated with an increased risk in stillbirth, supporting the Danish findings, and those of the meta-analysis described above ( Dhalwani *et al*., 2019). It must be noted, however, that women who were recorded as sole NRT users were categorized based on prescription data, and therefore misclassification is likely. In addition, stillbirth data was recorded from primary care records, and as most women give birth in a hospital setting, there is a chance that some cases may have been missed ( Dhalwani *et al*., 2019).

Conversely, an increased risk of stillbirth was found in women who used snus during pregnancy in Sweden. The study, which included data from 610,879 single births, showed that snus use was associated with an increased risk of stillbirth by approximately 60% compared to non-tobacco users. This increase in risk was more likely to manifest in preterm stillbirth (OR 2.1, 95% CI: 1.3-3.4), with term stillbirth risk only slightly increased (OR 1.3, 95% CI 0.76-2.1). There was no increase in risk of preeclampsia, antenatal bleeding, or small for gestational age associated with snus use ( Wikström *et al.,* 2010). England *et al*., however, found that snus use during pregnancy was associated with an increased risk of preterm birth and preeclampsia compared to non-tobacco users, although their cohort was much smaller (268 snus users). There was no information on the frequency of snus use in this study ( England *et al.*, 2003).

It is not clear as to why there are discrepancies in findings between studies in pregnant women who use snus, and those using NRT. It could be a nicotine dose effect, which is difficult to isolate from the published studies as most do not record data on the use frequency of either product.

## Lung development

Maternal smoking during pregnancy has been shown to result in adverse lung development in the offspring ( Chhabra *et al*., 2014). How much of this effect is due to nicotine is not known in humans, however, nicotinic receptors have been detected in several pulmonary cell types ( Filippini *et al*., 2012) suggesting that nicotine could bind and initiate downstream signaling during development.

### Human studies

A preliminary study into DNA methylation of aborted human fetal lungs, showed that placental cotinine concentration positively correlated with changes in several gene-specific methylation markers associated with various pathways, including asthma. However, the authors had no smoking history data for the mothers included in the study, and therefore the origin of the nicotine exposure could not be determined. Causality cannot be established from this observation, but the authors propose the use of differentially methylated regions as “a potential marker of injury in the developing lung exposed to nicotine *in utero*”. Further research is needed to establish the mechanisms behind lung injury in response to nicotine ( Chhabra *et al*., 2014).

### Animal studies

Three studies from the same research group have been carried out to investigate lung development in the rhesus monkey during maternal nicotine exposure ( Proskocil *et al*., 2005; Sekhon *et al*., 1999; Sekhon *et al*., 2001). This is a more similar model to human pregnancy in terms of placentation and uterine environment, in comparison to rodent models. Additionally, rodents are born in a more premature state compared to humans and monkeys, including the developmental stage of their lungs, making comparison even more challenging. In the first study by Sekhon *et al*., rhesus monkeys were dosed with nicotine at 1.0 mg/kg/day, using an osmotic pump, from day 26 of gestation until birth. Offspring were delivered by caesarean section on day 134 of gestation (equivalent to week 32 of human gestation). Levels of nicotine in the maternal serum, amniotic fluid and cord plasma were comparable to concentrations measured in pregnant human heavy smokers. Alveolar airspace was significantly increased, and corresponding surface area was decreased in nicotine-exposed offspring compared to controls. Alpha-7 nicotinic receptor subunits were identified in airway and arterial smooth muscle cells, fibroblasts surrounding the walls of airways as well as sub-mucous glands, airway-associated nerve fibers, and free pulmonary macrophages. Expression was significantly increased in cells lining the airway walls of nicotine-exposed offspring, suggesting that nicotine induced the upregulation of its receptor in the lung tissue, consistent with observations in the brain ( van de Kamp & Collins, 1994). The authors hypothesized that the increase in nicotinic receptors they observed in fibroblasts could lead to an increase in collagen deposition within the lung. In a follow-up experiment, using similar methods, the authors found that both the lung weight and lung volume was significantly reduced in rhesus monkey neonates that had been exposed to nicotine *in utero*. In addition, they found significant reductions in some lung function parameters, such as peak tidal expiratory volume, and an increase in pulmonary resistance. These results led the authors to conclude “These findings show that nicotine exposure during gestation leads to altered pulmonary function at birth” ( Sekhon *et al*., 2001).

In the study by Proskocil *et al*., offspring were delivered at day 160 of gestation (near term) by caesarean section. The lung function and histology of the offspring were measured; forced expiratory flow was significantly reduced in the nicotine-exposed group compared to controls, although forced expiratory volume was unchanged. The content of lung elastin was significantly reduced in both peripheral and central regions of the nicotine-treated offspring’s lungs; however, collagen was unchanged (contrary to the authors’ hypothesis in their previous paper). The changes shown were not mediated by any of the cytokines measured, leptin or cortisol ( Proskocil *et al*., 2005), however, some of the effects observed could be rescued by co-administration of vitamin C.

These papers support findings from the lungs of nicotine exposures in rodents (despite the drawbacks in rodent models) ( Maritz & Dennis, 1998; Wongtrakool *et al*., 2012) and some of the observations in the lungs of human offspring of smoking mothers ( Guerra *et al*., 2013; Sekhon *et al*., 2001). Although smoking is likely to have a more profound effect on human lung development than nicotine alone, it is possible that nicotine is responsible for some of the outcomes observed; however, the mechanisms remain unclear.

## Neurological effects

During fetal gestation, neurotransmitters act to orchestrate the development of specific neurological pathways ( Levitt *et al*., 1997). Nicotine is a neuroactive substance, which acts through nAChRs present from neural tube formation in the embryo ( Kinney *et al*., 1993). These induce changes to early brain morphology, however, as development continues, the morphological differences disappear. Despite the lack of long-term structural neurological changes in response to gestational nicotine exposure, there are additional effects, which are complex and difficult to map - they continue to change the trajectory of neurogenesis even when exposure has ceased. To make the picture more complex, nicotine has been shown to have both pro- and anti-apoptotic action on neurological tissue, as well as induce and prevent oxidative stress depending on the timing, cell type and concentration of exposure ( Pauly & Slotkin, 2008; Zelikoff *et al*., 2018). Additionally, stimulation of nAChRs led to the release of several neurotransmitters, including dopamine and serotonin, so the effect of nicotine exposure may be more far reaching than the acetylcholine pathways ( Pauly & Slotkin, 2008). Several studies suggest that the changes induced by nicotine in the developing brain lead to behavioral alterations that persist to adolescence and beyond ( Tiesler & Heinrich, 2014). There are so many exposures that could play a role in the uterine environment, as well as post-natal exposures, in addition to the wide-ranging descriptors that cover behavioral disorders, that it is impossible to pinpoint nicotine as a direct cause.

### Animal studies

In a study carried out in rhesus monkeys (a collaboration between Slotkin *et al.* and Sekhon *et al*., who carried out the lung development studies described above), the effects of maternal nicotine treatment on fetal brain nAChR binding, adenylyl cyclase activity and developmental biomarkers were examined ( Slotkin *et al*., 2005). Nicotine exposure resulted in an upregulation of α4β2 nAChRs across all regions of the fetal brain, similar to the levels observed in an adult smoker. α7 nAChRs were upregulated in the occipital cortex only. For all of the other parameters tested, the results were very specific to brain regions and there was no clear overriding pattern of effect; however, in addition to the changes in receptor induction, there was evidence to suggest decreased adenylyl cyclase activity in the occipital cortex and decreased cell numbers in the caudate region. Interestingly, the authors found an interaction with nicotine and both vitamin C and choline supplementation, which was protective in some instances and detrimental in others. They comment that as choline is recommended as a supplement during pregnancy in the USA, this could offer a mechanism that puts the offspring of smoking mothers at further risk of some brain abnormalities. Overall, the authors conclude that the findings in the rhesus monkey support the observations seen in developing rodent brain models, and are also likely to translate to human development ( Slotkin *et al*., 2005).

In a later study by the same group using the same rhesus monkey model, the effect of chronic nicotine exposure during gestation on serotonin (5-HT) in the brain was examined. Slotkin and colleagues found that levels of 5-HT were increased in in the brainstems of nicotine-exposed offspring, compared to non-exposed controls ( Slotkin *et al*., 2012). In a similar experiment in baboons, Duncan *et al.* found altered 5-HT binding in the medullae of nicotine-exposed offspring. This altered 5-HT activity may have implications regarding the cardiorespiratory control, supported by the observation of increased heart rate variation in the exposed animals ( Duncan *et al*., 2009).

### *In vitro* studies

Wang *et al.* used a newly developed ‘brain-on-a-chip’ model to investigate the effects of nicotine on human fetal brain development. Multi-cell-type brain organoids were grown *in vitro* and exposed to nicotine at 1 µM (similar to heavy human smoking) and 10 µM (above physiological concentration). The authors found that the 1 µM nicotine exposure led to premature neuronal differentiation increased neurite outgrowth and dysregulated forebrain and hindbrain cell populations. Although this model of human brain development is still a long way from mimicking development in the human fetus, it provides a considerable advance from traditional *in vitro* methods ( Wang *et al*., 2018).

## Metabolic disorders

### Animal studies

The effects of fetal and neonatal *in utero* nicotine exposure may not become apparent until later on in the life course of the offspring. Several studies in rats have described various adult outcomes including hypertension ( Xiao *et al*., 2008), increased adiposity ( Fan *et al*., 2016), and decreased pancreatic beta cell mass ( Bruin *et al*., 2008) after exposure to nicotine during gestation and lactation. The mechanisms behind these associations are currently unclear, but are likely to include increased oxidative stress ( Bruin *et al*., 2008) and could provide a link to the effects of smoking during human pregnancy to the increased prevalence of hypertension ( Blake *et al*., 2000) and type 2 diabetes ( Bruin *et al*., 2010). Although nicotine is a possible cause, there are no studies investigating the effects of *in utero* nicotine on measures of impaired cardiovascular or metabolic outcomes in humans.

In conclusion, although the use of e-cigarettes have been estimated to be up to 95% less toxic compared to conventional cigarettes ( McNeill *et al*., 2015), and NRTs to cause very low risk of harm ( Niaura, 2016), pregnancy provides a different paradigm. The weight of the evidence provided by animal studies suggests that nicotine itself is likely to be detrimental to fetal lung ( Proskocil *et al*., 2005) and brain development, possibly at concentrations lower than the threshold needed to cause a reduction in birth weight ( Slotkin *et al*., 2005). Long-term changes to nicotinic receptor distribution in the offspring, and the potential raft of associated neurological alterations ( Pauly & Slotkin, 2008) make the downstream effects of *in utero* nicotine exposure broad and difficult to isolate in experimental models.

## Limitations

This narrative review was not carried out as a systematic review. The search results from PubMed omitted many well known and important studies, which were included from other sources. The PubMed search did not provide the majority of the literature cited. Future reviews on this topic should ideally be carried out systematically, using multiple sources. We cannot exclude the possibility that further relevant studies may not have been included.

## Conclusion

Literature investigating the effects of nicotine exposure in relation to cardiovascular risks, cancer risks and reproductive outcomes in humans is limited. As a result, it is difficult to pinpoint the role of nicotine as a single risk factor in the manifestation of these multifactorial conditions. No single risk factor can give rise to multifactorial disease by itself because, as a minimum, both the environment and endogenous risk factors must interact. Rothman and Greenland state, “[T]he importance of multicausality is that most identified causes are neither necessary nor sufficient to produce disease” ( Rothman & Greenland, 2005).

While the mechanisms by which nicotine acts on the cardiovascular system are well established and can be observed *in vivo* after nicotine administration, the long-term consequences of these responses are not known. Currently, the literature suggests that in consumers with no underlying cardiovascular pathology, there is no increased risk due to nicotine exposure.

There is general consensus that nicotine is not a direct or complete carcinogen, however, it remains to be established whether it has a role in cancer propagation and metastasis. Experimental molecular biology can demonstrate possible cancer progression pathways that may be brought about in response to nicotine, but these have not been investigated in cases of human cancer.

Further studies are needed to determine whether nicotine is detrimental to fertility in humans. Nevertheless, the results from animal studies indicate that nicotine has many and wide-ranging effects on fetal development.

We are now entering a period of rapid change in smoking behavior, from conventional combustible products to new ENDS, and extensive scientific research is needed to characterize the effects of nicotine exposure in humans more thoroughly.

## Data availability

### Underlying data

All data underlying the results are available as part of the article and no additional source data are required.

### Extended data

Figshare: Extended Data - Biological Effects of Nicotine Exposure: A narrative review of the scientific literature. https://doi.org/10.6084/m9.figshare.9638234.v1 ( Price & Martinez, 2019)

This project contains the following extended data:

- Paper Screening Master File.xlsx (Spreadsheet of full list of articles returned by search)
- Supplementary Methods.docx (Search methods, criteria and results for a non-systematic literature review)

Data are available under the terms of the Creative Commons Zero “No rights reserved” data waiver (CC0 1.0 Public domain dedication).
