# Supplementary material for: Cardiovascular, carcinogenic and reproductive effects of nicotine exposure: A narrative review of the scientific literature
Source: F1000Res. 2020 Jan 9;8:1586. Originally published 2019 Sep 4. [Version 2] doi: 10.12688/f1000research.20062.2 (PMC7308884; doi:10.12688/f1000research.20062.2)
Supplement: Peer review history

# Peer review: Reviewer response for version 2

**DOI:** 10.5256/f1000research.24079.r63761

**Published:** 2020-06-17

**Authors:** Alison Holloway

**Affiliations:** 1 Obstetrics and Gynecology, McMaster University, Hamilton, Canada

In the section on cardiovascular outcomes, I think it is important to address the fact that most of the studies looking at the effects of nicotine have been done in adults. The effects of nicotine on these same outcomes in adolescents and youth is not well known and may in fact be different.

 It is somewhat surprising that there is no section describing birth outcomes in animal studies as there have been a number of rodent studies done with nicotine alone and more recently studies looking at the effects of ENDS use during pregnancy in rodent models. Many of these studies have described pregnancy outcomes including birthweight and live birth index (a surrogate for stillbirth).

Is the review written in accessible language?

Yes

Are all factual statements correct and adequately supported by citations?

Yes

Are the conclusions drawn appropriate in the context of the current research literature?

Yes

Is the topic of the review discussed comprehensively in the context of the current literature?

Partly

Reviewer Expertise:

Nicotine exposure during pregnancy and postnatal health.

I confirm that I have read this submission and believe that I have an appropriate level of expertise to confirm that it is of an acceptable scientific standard.

# Peer review: Reviewer response for version 2

**DOI:** 10.5256/f1000research.24079.r58432

**Published:** 2020-01-09

**Authors:** Bernd Mayer

**Affiliations:** 1 Department of Pharmacology and Toxicology Institute of Pharmaceutical Sciences, Karl-Franzens-University Graz, Graz, Austria

The authors have considered most of the points I had raised, so I wish to confirm the Approved status.

Is the review written in accessible language?

Yes

Are all factual statements correct and adequately supported by citations?

Yes

Are the conclusions drawn appropriate in the context of the current research literature?

Yes

Is the topic of the review discussed comprehensively in the context of the current literature?

Yes

Reviewer Expertise:

Active research in cardiovascular pharmacology, longstanding interest in nicotine.

I confirm that I have read this submission and believe that I have an appropriate level of expertise to confirm that it is of an acceptable scientific standard.

# Peer review: Reviewer response for version 1

**DOI:** 10.5256/f1000research.22028.r56498

**Published:** 2019-12-03

**Authors:** Bernd Mayer

**Affiliations:** 1 Department of Pharmacology and Toxicology Institute of Pharmaceutical Sciences, Karl-Franzens-University Graz, Graz, Austria

This is a comprehensive review of the most prominent and widely discussed biological effects of nicotine that could be relevant for users of new nicotine products, in particular, electronic cigarettes and tobacco heating systems. The article is well balanced, covering the relevant literature, and urgently needed to distinguish the effects of nicotine from the well established detrimental effects of tobacco smoke. I highly recommend the indexing of the paper, but the authors may wish to address the following points to further increase the impact of their work.

**General**

1. Press releases on publications describing potential consequences of vaping, with or without nicotine, tend to confuse media representatives, policymakers and the public by mixing up human data from clinical studies with results from rodents exposed to vapor and *in vitro *data. I appreciate the authors' efforts to clearly distinguish between these categories, which differ largely in their relevance for consumers. Although the current paper describes the discrepancies between animal and human studies on several occasions, I missed a few paragraphs explicitly discussing the striking lack of translation of rodent results to humans with respect to the biology of nicotine. Such cautionary notes may help to prevent the widespread over interpretation of experimental results that are irrelevant for human consumers.
2. While the harmful effects of nicotine are comprehensively discussed, the authors do not even mention potential beneficial effects, such as protection from the development of inflammatory brain diseases and ulcerative colitis as well as improvement of cognitive funtions and AD(H)S. Although controversial, these benefits of nicotine appear to be better documented than some of the harms that are extensively described in the article.

**Specific Points**

Acute nicotine toxicity

1. The authors should provide the suggested value of the revised lethal oral nicotine dose for adults (0.5 - 1g; ref. Mayer (2014) ^1^). In the paragraph on the toxicity of patches, it is stated "although not ingested". This is misleading because the dermal bioavailability of nicotine is much higher than oral.
2.  It should be mentioned that the number of calls counted by poison centers does not necessarily reflect the number of incidences of real poisoning. I suggest providing the numbers concerning toilet cleaners (or varenicline) for comparison.

Cardiovascular effects

1. The cardiovascular effects of nicotine may be partially due to noradrenaline release from sympathetic nerve terminals (as illustrated in Fig. 1), but activation of nicotinic receptors expressed in vegetative ganglia should also be mentioned.
2. There is no compelling evidence for an increased blood pressure of smokers (statement on p. 5, left panel, 2nd paragraph). Most epidemiological studies indicate either no difference or lower blood pressure of smokers as compared to non-smokers. For relatively recent studies see e.g.: Akbarour *et al*. (2019) ^2^; Li *et al*. (2017) ^3^; Papathanasiou *et al*. (2015) ^4^; Linneberg *et al*. (2015) ^5^; Li *et al*. (2010) ^6^.
3. The part on endothelial function should be updated by including two very recent studies on the effects of vaping with nicotine on flow-mediated dilation. While Kuntic *et al*. (2019) ^7^ reported on acute endothelial dysfunction of smokers 15 minutes after vaping, George *et al*. (2019) ^8^ observed significant improvement of FMD within one month of switching from smoking to electronic cigarettes.

Is the review written in accessible language?

Yes

Are all factual statements correct and adequately supported by citations?

Yes

Are the conclusions drawn appropriate in the context of the current research literature?

Yes

Is the topic of the review discussed comprehensively in the context of the current literature?

Yes

Reviewer Expertise:

Active research in cardiovascular pharmacology, longstanding interest in nicotine.

I confirm that I have read this submission and believe that I have an appropriate level of expertise to confirm that it is of an acceptable scientific standard.

## References

[1] : How much nicotine kills a human? Tracing back the generally accepted lethal dose to dubious self-experiments in the nineteenth century. *Arch Toxicol*.2014;88(1) : 10.1007/s00204-013-1127-0 5-7 10.1007/s00204-013-1127-0 24091634PMC3880486

[2] : Relationship between lifestyle pattern and blood pressure - Iranian national survey. *Sci Rep*.2019;9(1) : 10.1038/s41598-019-51309-3 15194 10.1038/s41598-019-51309-3 31645585PMC6811561

[3] : The association between smoking and blood pressure in men: a cross-sectional study. *BMC Public Health*.2017;17(1) : 10.1186/s12889-017-4802-x 797 10.1186/s12889-017-4802-x 29017534PMC5634904

[4] : Association of high blood pressure with body mass index, smoking and physical activity in healthy young adults. *Open Cardiovasc Med J*.2015;9: 10.2174/1874192401509010005 5-17 10.2174/1874192401509010005 25834651PMC4378027

[5] : Effect of Smoking on Blood Pressure and Resting Heart Rate: A Mendelian Randomization Meta-Analysis in the CARTA Consortium. *Circ Cardiovasc Genet*.2015;8(6) : 10.1161/CIRCGENETICS.115.001225 832-41 10.1161/CIRCGENETICS.115.001225 26538566PMC4684098

[6] : Effects of cigarette smoking on blood pressure stratified by BMI in Mongolian population, China. *Blood Press*.2010;19(2) : 10.3109/08037050903516300 92-7 10.3109/08037050903516300 20070251

[7] : Short-term e-cigarette vapour exposure causes vascular oxidative stress and dysfunction: evidence for a close connection to brain damage and a key role of the phagocytic NADPH oxidase (NOX-2). *Eur Heart J*.2019; 10.1093/eurheartj/ehz772 10.1093/eurheartj/ehz772 31715629PMC7340357

[8] : Cardiovascular Effects of Switching From Tobacco Cigarettes to Electronic Cigarettes. *J Am Coll Cardiol*.2019; 10.1016/j.jacc.2019.09.067 10.1016/j.jacc.2019.09.067 30621938

# Response

**Published:** 2019-12-20

**Authors:** Leonie Price

**Affiliations:** Japan Tobacco International, Switzerland

Dear Dr. Mayer,

 Thank you for taking the time to peer review our paper and provide such positive feedback. Please find below our response to your general and specific suggestions point-by-point. We hope you find our changes satisfactory.

 Best regards,

 Leonie Price and Javier Martinez

**General points**

- Press releases on publications describing potential consequences of vaping, with or without nicotine, tend to confuse media representatives, policymakers and the public by mixing up human data from clinical studies with results from rodents exposed to vapor and in vitro data. I appreciate the authors' efforts to clearly distinguish between these categories, which differ largely in their relevance for consumers. Although the current paper describes the discrepancies between animal and human studies on several occasions, I missed a few paragraphs explicitly discussing the striking lack of translation of rodent results to humans with respect to the biology of nicotine. Such cautionary notes may help to prevent the widespread over interpretation of experimental results that are irrelevant for human consumers.
- *We agree; however, we have not added any additional content regarding issues related to animal models of human disease. We hope that by dividing the manuscript into sections addressing the various in vivo and in vitro studies as well as describing differences between them enough to highlight the limitations. Unfortunately, inaccurate media representation of scientific data is an issue that spans all scientific fields. We hope that the more scientists engage with journalists, the quicker this problem can be eliminated.*

 

- While the harmful effects of nicotine are comprehensively discussed, the authors do not even mention potential beneficial effects, such as protection from the development of inflammatory brain diseases and ulcerative colitis as well as improvement of cognitive functions and AD(H)S. Although controversial, these benefits of nicotine appear to be better documented than some of the harms that are extensively described in the article.
- *We have added reference to ulcerative colitis in the context of inflammation. As you rightly point out, the positive effects of nicotine are controversial, and therefore need to be assessed with caution. We have not addressed any of the cognitive effects, as this subject falls beyond the scope of the paper; however, this could be an important topic for future work.*

**Specific Points**

**Acute nicotine toxicity**

- The authors should provide the suggested value of the revised lethal oral nicotine dose for adults (0.5 - 1g; ref. Mayer (2014)1). In the paragraph on the toxicity of patches, it is stated "although not ingested". This is misleading because the dermal bioavailability of nicotine is much higher than oral.

- *Thank you – we agree this was not clear and have deleted this sentence.*

Line 71

  

- It should be mentioned that the number of calls counted by poison centers does not necessarily reflect the number of incidences of real poisoning. I suggest providing the numbers concerning toilet cleaners (or varenicline) for comparison.
- *Thank you for highlighting this. We have added some context to the poison statistics in the form of data from the same US poison centers used in the Chatham-Stephens study, as well as adding your excellent point.*

Lines 85-92

**Cardiovascular effects**

- The cardiovascular effects of nicotine may be partially due to noradrenaline release from sympathetic nerve terminals (as illustrated in Fig. 1), but activation of nicotinic receptors expressed in vegetative ganglia should also be mentioned.
- *Agree – we have now mentioned this mechanism.*

Lines 98-100

  

- There is no compelling evidence for an increased blood pressure of smokers (statement on p. 5, left panel, 2nd paragraph). Most epidemiological studies indicate either no difference or lower blood pressure of smokers as compared to non-smokers. For relatively recent studies see e.g.: Akbarour et al. (2019)2; Li et al. (2017)3; Papathanasiou et al. (2015)4; Linneberg et al. (2015)5; Li et al. (2010)6.
- *You are, of course, correct. The wording of this paragraph has been updated to more accurately describe the participants in the Farsalinos study, rather than smokers in general.*

Lines 151-153

  

- The part on endothelial function should be updated by including two very recent studies on the effects of vaping with nicotine on flow-mediated dilation. While Kuntic et al. (2019)7 reported on acute endothelial dysfunction of smokers 15 minutes after vaping, George et al. (2019)8 observed significant improvement of FMD within one month of switching from smoking to electronic cigarettes.
- *These references have now been added.*

Lines 381-403

# Peer review: Reviewer response for version 1

**DOI:** 10.5256/f1000research.22028.r53455

**Published:** 2019-09-30

**Authors:** Lukasz Antoniewicz

**Affiliations:** 1 Division of Internal Medicine, Department of Clinical Sciences, Danderyd University Hospital, Karolinska Institutet, Stockholm, Sweden

This narrative review is interestingly written. However, several major points need to be addressed prior to acceptance.

 The title "Biological effects of nicotine exposure“ is misleading as the article is focusing on cardiovascular, carcinogenic and reproductive effects of nicotine. The wide spectrum of neurological effects and especially addictive effects is not mentioned or discussed.

**Methods:**

 The biggest methodological issues are the inclusion criteria for this review. As stated, ten search terms were used in PubMed to carry out literature search. Additionally, relevant studies and studies prior to 2009 referenced in the returned papers were included. Additional relevant studies from JTI databases were also added. In the Supplementary Methods flowchart, initially 2301 references were found. 33 were finally included from this source. However, 31 papers were included by reference and 58 from the JTI-database. The majority of cited papers were included without standardized search criteria.

**Introduction:**

 The authors state that “It is already understood that nicotine is not a major contributing factor to the diseases associated with tobacco smoking“ referring to ( Benowitz *et al*., 1998; Gottlieb & Zeller, 2017; Royal College of Physicians, 2016). Gottlieb *et al*. is a short commentary, itself not referring to any data supporting this statement. It states however "Nicotine is, however, responsible for getting smokers addicted to cigarette smoking in the first place - usually while they are children or young adults and their brains are still developing - and keeping them addicted long-term". As mentioned before, this is omitted in the entire work.

 Benowitz *et al. *is a very good review on nicotine research published 1998. A more recent publication would be feasible.

 The Royal College of Physicians states "The safety of NRT products, which have typically been used for days or weeks in the context of an attempt to quit smoking, is well established …. with no evidence of any increase in the risk of heart attack, stroke or death". The introduction, stating that "it is already understood that nicotine is not a major contributing factor” is in my opinion not supported by the referenced articles.

 “Extensive analysis into nicotine as a pharmacological molecule has already been carried out”. In the listed references, West *et al.* and Baraona *et al.* did not carry out any investigations on nicotine as a pharmacological molecule.

**Acute nicotine toxicity**

 The authors compare the ingested dose of 60 mg to the transdermally applied dose of 64 mg. The line “(interestingly higher than the “lethal” dose, although not ingested)” is obsolete as the routes of nicotine administration lead to completely different serum-nicotine-levels.

**Cardiovascular effects**

 In the end the authors state “Although there are immediate pharmacological effects of nicotine on cardiovascular parameters, there is little evidence to suggest that there is an increase in risk to long-term cardiovascular health as a result of nicotine exposure from either NRT or e-cigarettes.” That final statement may clearly be misread as no long-term studies (longer than 10 years of sole e-cigarette or NRT use) exist. In several paragraphs the authors discuss the effects of Swedish snus – to discuss long-term nicotine effects. Indeed several important studies suggest that long-term snus use may be associated with higher mortality when developing cardiovascular disease, the development of diabetes and endothelial dysfunction ^1^^,^^2^^,^^3^^,^^4^^,^^5^. Especially the study by Arefalk *et al*. (2014) ^3^ needs to be addressed.

**Thrombosis, inflammation and atherosclerosis**

 The role of nicotine in the propagation of atherosclerosis and diabetic retinopathy should be discussed more thoroughly ^6^^,^^7^^,^^8^^,^^9^. Additionally, a summary of this topic should be added at the end of this paragraph.

**Pregnancy and offspring**

 Effects of snus use and pregnancy should be discussed as well. For example: ^10^.

Is the review written in accessible language?

Yes

Are all factual statements correct and adequately supported by citations?

Partly

Are the conclusions drawn appropriate in the context of the current research literature?

Partly

Is the topic of the review discussed comprehensively in the context of the current literature?

Partly

Reviewer Expertise:

Cardiovascular Research. Effects of nicotine, e-cigarettes, tobacco and snus on endothelial function.

I confirm that I have read this submission and believe that I have an appropriate level of expertise to state that I do not consider it to be of an acceptable scientific standard, for reasons outlined above.

## References

[1] : Smokeless tobacco (snus) and risk of heart failure: results from two Swedish cohorts. *Eur J Prev Cardiol*.2012;19(5) : 10.1177/1741826711420003 1120-7 10.1177/1741826711420003 21828223

[2] : High consumption of smokeless tobacco ("snus") predicts increased risk of type 2 diabetes in a 10-year prospective study of middle-aged Swedish men. *Scand J Public Health*.2012;40(8) : 10.1177/1403494812459814 730-7 10.1177/1403494812459814 23117209

[3] : Discontinuation of smokeless tobacco and mortality risk after myocardial infarction. *Circulation*.2014;130(4) : 10.1161/CIRCULATIONAHA.113.007252 325-32 10.1161/CIRCULATIONAHA.113.007252 24958793

[4] : Non-Smoking Tobacco Affects Endothelial Function in Healthy Men in One of the Largest Health Studies Ever Performed; The Nord-Trøndelag Health Study in Norway; HUNT3. *PLoS One*.2016;11(8) : 10.1371/journal.pone.0160205 e0160205 10.1371/journal.pone.0160205 27490361PMC4974005

[5] : Smokeless tobacco and the risk of stroke. *Epidemiology*.2008;19(6) : 10.1097/EDE.0b013e3181878b33 794-9 10.1097/EDE.0b013e3181878b33 18854704

[6] : Aortic remodelling in chronic nicotine-administered rat. *Korean J Physiol Pharmacol*.2014;18(5) : 10.4196/kjpp.2014.18.5.411 411-8 10.4196/kjpp.2014.18.5.411 25352761PMC4211125

[7] : Long-term nicotine exposure induces dysfunction of mouse endothelial progenitor cells. *Exp Ther Med*.2017;13(1) : 10.3892/etm.2016.3916 85-90 10.3892/etm.2016.3916 28123473PMC5244833

[8] : Nicotine promotes vascular endothelial growth factor secretion by human trophoblast cells under hypoxic conditions and improves the proliferation and tube formation capacity of human umbilical endothelial cells. *Reprod Biomed Online*.2017;34(4) : 10.1016/j.rbmo.2016.12.014 406-413 10.1016/j.rbmo.2016.12.014 28109704

[9] : Nicotine accelerates diabetes-induced retinal changes. *Curr Eye Res*.2015;40(4) : 10.3109/02713683.2014.924147 368-77 10.3109/02713683.2014.924147 24911405PMC4451833

[10] : Maternal use of Swedish snuff (snus) and risk of stillbirth. *Epidemiology*.2010;21(6) : 10.1097/EDE.0b013e3181f20d7e 772-8 10.1097/EDE.0b013e3181f20d7e 20805750

# Response

**Published:** 2019-10-01

**Authors:** Leonie Price

**Affiliations:** Japan Tobacco International, Switzerland

Dear Dr. Antoniewicz,

Thank you very much for taking the time to review our paper. We appreciate all your comments and will be more than happy to address them in the coming days, making amendments to the manuscript as appropriate. We strongly believe that paper will be significantly improved thanks to your input.

Kind regards,

Leonie Price and Javier Martinez

# Response

**Published:** 2019-12-20

**Authors:** Leonie Price

**Affiliations:** Japan Tobacco International, Switzerland

Dear Dr. Antoniewicz,

 Thank you once again for taking the time to review our paper. We were advised to wait until our second peer review before making any changes to the manuscript, hence the delay in response. Please find below our reply to your suggestions point-by-point. We hope you find our changes satisfactory.

 Best regards,

 Leonie Price and Javier Martinez

**Point-by-point response**

- The title "Biological effects of nicotine exposure” is misleading as the article is focusing on cardiovascular, carcinogenic and reproductive effects of nicotine. The wide spectrum of neurological effects and especially addictive effects is not mentioned or discussed.
- *Well noted, the title has been updated to reflect the specific areas covered in the review.*

Page 1

**Methods:**

- The biggest methodological issues are the inclusion criteria for this review. As stated, ten search terms were used in PubMed to carry out literature search. Additionally, relevant studies and studies prior to 2009 referenced in the returned papers were included. Additional relevant studies from JTI databases were also added. In the Supplementary Methods flowchart, initially 2301 references were found. 33 were finally included from this source. However, 31 papers were included by reference and 58 from the JTI-database. The majority of cited papers were included without standardized search criteria.
- *We agree – We found the publications list returned from the PubMed search disappointing, especially due to the large number of relevant and important papers we had available in our databases. We felt it necessary to include these publications despite them not appearing in the PubMed list. We hope to have now addressed your concern by making this point clearer in the manuscript through expanding the methods and limitations sections.*

Lines 37-42 and 1047-1052

**Introduction:**

- The authors state that “It is already understood that nicotine is not a major contributing factor to the diseases associated with tobacco smoking” referring to (Benowitz et al., 1998; Gottlieb & Zeller, 2017; Royal College of Physicians, 2016). Gottlieb et al. is a short commentary, itself not referring to any data supporting this statement. It states however "Nicotine is, however, responsible for getting smokers addicted to cigarette smoking in the first place - usually while they are children or young adults and their brains are still developing - and keeping them addicted long-term". As mentioned before, this is omitted in the entire work.
- *You are correct, the addictive nature of nicotine was deliberately omitted from the review as the focus was on the specific areas biological effect outlined. The secondary effects of nicotine addiction, contributing to prolonged smoking and the associated health implications are related to the effects of inhaled combusted tobacco, and do not manifest as a result of nicotine exposure alone. Having said this, we have now updated the wording of this sentence. It now reads as follows: “Various reviews of published scientific literature and Public Health statements have concluded that despite its addictive nature, nicotine is not a major contributing factor to diseases associated with tobacco smoking (Benowitz et al., 2016; Gottlieb & Zeller, 2017; Royal College of Physicians, 2016; Surgeon General, 2014)”.*

Lines 5-10

  

- Benowitz et al. is a very good review on nicotine research published 1998. A more recent publication would be feasible.
- *This citation has been updated.*

Line 9

  

- The Royal College of Physicians states "The safety of NRT products, which have typically been used for days or weeks in the context of an attempt to quit smoking, is well established …. with no evidence of any increase in the risk of heart attack, stroke or death". The introduction, stating that "it is already understood that nicotine is not a major contributing factor” is in my opinion not supported by the referenced articles.
- *Addressed as part of Introduction point 1. The sentence now reads “Various reviews of published scientific literature and Public Health statements have concluded that despite its addictive nature, nicotine is not a major contributing factor to diseases associated with tobacco smoking (Benowitz et al., 2016; Gottlieb & Zeller, 2017; Royal College of Physicians, 2016; Surgeon General, 2014)”. The Royal College of Physicians do indeed state as quoted; however, the overall conclusions of their publication say the following: “People smoke because they are addicted to nicotine, but are harmed by other constituents of tobacco smoke. Provision of the nicotine that smokers are addicted to without the harmful components of tobacco smoke can prevent most of the harm from smoking”.*

Lines 5-10

  

- “Extensive analysis into nicotine as a pharmacological molecule has already been carried out”. In the listed references, West *et al.* and Baraona *et al.* did not carry out any investigations on nicotine as a pharmacological molecule.
- *We have made it clear that the Barona and West articles review the research and did not carry out the studies.*

Line 19.

**Acute nicotine toxicity**

- The authors compare the ingested dose of 60 mg to the transdermally applied dose of 64 mg. The line “(interestingly higher than the “lethal” dose, although not ingested)” is obsolete as the routes of nicotine administration lead to completely different serum-nicotine-levels.
- *We have deleted this sentence.*

Line 71

**Cardiovascular effects**

- In the end the authors state “Although there are immediate pharmacological effects of nicotine on cardiovascular parameters, there is little evidence to suggest that there is an increase in risk to long-term cardiovascular health as a result of nicotine exposure from either NRT or e-cigarettes.” That final statement may clearly be misread as no long-term studies (longer than 10 years of sole e-cigarette or NRT use) exist. In several paragraphs the authors discuss the effects of Swedish snus – to discuss long-term nicotine effects. Indeed several important studies suggest that long-term snus use may be associated with higher mortality when developing cardiovascular disease, the development of diabetes and endothelial dysfunction1,2,3,4,5. Especially the study by Arefalk et al. (2014) needs to be addressed.
- *We understand how our wording could be misinterpreted here and have added clarity to this section.*

Lines 349-354

- *The publications by Arefalk et al. (2012), Arefalk et al. (2014), Skaug et al. (2016) have been addressed.*

Lines 260-274 and 191-199.

- *Hergens et al. 2008 has been addressed alongside the publications referring to stroke risk already included, in the Thrombosis section.*

Lines 367-371

- *The suggested paper by Östenson et al. has not been included, as diabetes risk is beyond the scope of the paper. We agree that this could be an important topic for future work.*
- *None of the additional papers change the conclusions reached in the review as published.*

 

**Thrombosis, inflammation and atherosclerosis**

- The role of nicotine in the propagation of atherosclerosis and diabetic retinopathy should be discussed more thoroughly 6,7,8,9. Additionally, a summary of this topic should be added at the end of this paragraph.
- *Thank you – Zainalabidin et al. and Li et al. have now been addressed in this section.*

Lines 437-455 and 466-472

- *We have not addressed Boretsky et al. as diabetes is beyond the scope of the review. As above, we agree that this could be an important topic for future work. We have also not included Zhao et al., as this study investigated nicotine as a potential protective substance against preeclampsia in vitro using HUVEC cells. Published studies in humans have suggested that despite a protective effect of cigarette smoking on the risk of developing preeclampsia in pregnancy, the effect is lost in pregnant women who use snus (Wikström et al., 2010; England et al., 2003). Therefore, the current hypotheses suggest that it is a substance other than nicotine in cigarette smoke that is responsible for the protective effect described epidemiologically. It is unclear, therefore, whether the in vitro findings of Zhao’s study offer any further insight into the clinical outcomes in humans.*
- *The section has been summarised.*

Lines 473-483

**Pregnancy and offspring**

- Effects of snus use and pregnancy should be discussed as well. For example:10
- *Agree. We have included the suggested reference as well as England et al. 2003.*

Lines 897-911
